# Modulation of Global Low-Frequency Motions Underlies Allosteric Regulation: Demonstration in CRP/FNR Family Transcription Factors

**DOI:** 10.1371/journal.pbio.1001651

**Published:** 2013-09-10

**Authors:** Thomas L. Rodgers, Philip D. Townsend, David Burnell, Matthew L. Jones, Shane A. Richards, Tom C. B. McLeish, Ehmke Pohl, Mark R. Wilson, Martin J. Cann

**Affiliations:** 1 Biophysical Sciences Institute, Durham University, Durham, United Kingdom; 2 Department of Chemistry, Durham University, Durham, United Kingdom; 3 School of Biological and Biomedical Sciences, Durham University, Durham, United Kingdom; 4 Department of Physics, Durham University, Durham, United Kingdom; Stanford University, United States of America

## Abstract

Allostery is a fundamental process by which ligand binding to a protein alters its activity at a distinct site. There is growing evidence that allosteric cooperativity can be communicated by modulation of protein dynamics without conformational change. The mechanisms, however, for communicating dynamic fluctuations between sites are debated. We provide a foundational theory for how allostery can occur as a function of low-frequency dynamics without a change in structure. We have generated coarse-grained models that describe the protein backbone motions of the CRP/FNR family transcription factors, CAP of *Escherichia coli* and GlxR of *Corynebacterium glutamicum*. The latter we demonstrate as a new exemplar for allostery without conformation change. We observe that binding the first molecule of cAMP ligand is correlated with modulation of the global normal modes and negative cooperativity for binding the second cAMP ligand without a change in mean structure. The theory makes key experimental predictions that are tested through an analysis of variant proteins by structural biology and isothermal calorimetry. Quantifying allostery as a free energy landscape revealed a protein “design space” that identified the inter- and intramolecular regulatory parameters that frame CRP/FNR family allostery. Furthermore, through analyzing CAP variants from diverse species, we demonstrate an evolutionary selection pressure to conserve residues crucial for allosteric control. This finding provides a link between the position of CRP/FNR transcription factors within the allosteric free energy landscapes and evolutionary selection pressures. Our study therefore reveals significant features of the mechanistic basis for allostery. Changes in low-frequency dynamics correlate with allosteric effects on ligand binding without the requirement for a defined spatial pathway. In addition to evolving suitable three-dimensional structures, CRP/FNR family transcription factors have been selected to occupy a dynamic space that fine-tunes biological activity and thus establishes the means to engineer allosteric mechanisms driven by low-frequency dynamics.

## Abstract 2

Allostery in bacterial transcription factors arises from changes in global low-frequency protein dynamics. Amino acids that regulate low-frequency dynamics are identified and seen to be evolutionarily conserved.

## Author Summary

Allostery is a process by which a molecule binding to one site of a protein alters the activity of the protein at another site. Allostery is typically thought to occur through a change in protein structure, but there is now clear evidence that the dynamic properties of a protein can also regulate allostery without a change in overall conformation. Here we examine two members of a large family of bacterial transcription factors and provide a mechanism to describe the allosteric binding of their activating ligands. We demonstrate, in these systems, that allostery arises as a natural consequence of changes in global low-frequency protein fluctuations on ligand binding. We further demonstrate that the higher dimensional parameter space that describes all potential variant transcription factors can be reduced to a two-dimensional free energy landscape that determines the key molecular parameters that predominantly regulate allostery. We additionally show that the amino acids we determine as contributing sensitively to allosteric control tend to be conserved in diverse bacteria; thus we identify a link between residues that contribute to low-frequency fluctuations and evolutionary selection pressures.

## Introduction

Small regulatory molecules frequently bind proteins at regions remote from the active site. These allosteric events can switch proteins between inactive and active states [1]. Knowledge of the molecular basis of allostery derives from a wealth of theoretical and experimental studies and traditionally describes the process in terms of conformational change within the protein [2],[3]. Combinations of X-ray crystallography and NMR have permitted analysis of the ligand binding sites, intermolecular interactions, and conformational fluctuations that underpin diverse allosteric systems [4],[5]. There is also considerable evidence that allosteric cooperativity can be communicated between distant sites on proteins through modulation of their dynamic properties, even in cases where that are no structural changes between the ligand bound (holo) and unbound (apo) forms [6]–[12]. Since the original identification, by Cooper and Dryden [4], of this alternative route of “allostery without conformational change,” there has been considerable debate over the mechanisms by which dynamic fluctuations are communicated between allosterically coupled sites on proteins.

One hypothesis for fluctuation-induced allostery is that binding modifies the structure of the thermally excited global normal modes and thence the coupling interaction between cooperative elements. This in turn affects the structural ensemble of the distant sites and so the free energy of binding [13]–[15]. Another view maintains that physically connected pathways of excited or repressed dynamics, coupled along their trajectories, connect allosteric sites [16]–[18]. Here we propose the hypothesis that the normal modes of protein structural motion, large-scale motions dispersed across the entire protein, are important carriers of the allosteric signal and act without requiring structural change. Previous studies of the normal modes have demonstrated that conformational transitions in proteins, including those that underpin allosteric regulation dependent on conformational change, are well described by one or a few low-frequency modes [19]–[25]. The normal modes, however, can also be used to describe the whole spectrum of internal fluctuations of a protein around a mean structure. The low-frequency global modes, in particular, can involve entire protein domains. Alteration of the normal modes might therefore be communicated to distant sites of a protein as a change in the degree of motion around a mean structure without overall conformational change. Global low-frequency fluctuation therefore represents an alternative theoretical approach to allosteric communication that does not depend upon conformational change. An important consequence of this alternative mechanism of allosteric communication is that it can be captured by coarse-grained representations and models, such as the elastic network model (ENM). Here we develop this theory, and the validity of a coarse-grained model approach, through a computational and experimental study of the homodimeric CRP/FNR family transcription factors Catabolite Activator Protein (CAP) of *Escherichia coli* and GlxR of *Corynebacterium glutamicum*.

CAP is a 210-amino-acid transcription factor that binds cAMP generated by adenylyl cyclase in response to the phosphorylated form of Enzyme IIA^Glc^ (phosphorylated in response to the phosphoenolpyruvate-carbohydrate phosphotransferase system) [26],[27]. cAMP-bound CAP regulates the transcription of over 100 genes crucial for carbon utilization through its binding to a specific promoter region and recruitment of RNA polymerase [28]. Previous studies of the ligand binding domain of CAP demonstrated negative cooperativity between cAMP binding sites in the absence of structural change within this domain [10]. The observed negative cooperativity in this isolated domain occurs through a conformational entropic penalty for binding the second molecule of cAMP, but there is no mechanistic description for how such a phenomenon can occur in the full-length protein. Seven of eight CAP mutants previously examined showed a direct correlation between ΔΔ*G* and the adiabatic compressibility (β_s_°) where proteins with a higher β_s_° (reflecting increased structural flexibility in solution) demonstrated enhanced negative cooperativity [29]. While it is therefore reasonable to hypothesize a role for protein dynamics in allostery in CAP, there is no conceptual framework to describe how these changes in motion might arise, how they contribute to allostery, and how a resulting theory might translate to related molecules. CAP is therefore a suitable model system for a theoretical and experimental investigation of the contribution of the normal modes to allostery.

Here we propose that changes to global low-frequency protein backbone fluctuations are carriers of an allosteric signal in CAP and present this in the context of a significant new quantitative theory for allosteric coupling. We produce coarse-grained models that describe global low-frequency protein backbone motions of CAP and show a strong correlation between negative cooperativity for cAMP and modulation of the delocalised normal modes on ligand binding without a requirement for a spatially distinct physical pathway or conformational change. We demonstrate experimentally that altered connectivity between backbone elements in CAP can give predictable alterations to cooperativity for cAMP binding through altered mode amplitudes. We further demonstrate a broader applicability for this theory using an additional CRP/FNR family transcription factor, GlxR of *C. glutamicum*. We unite our findings for CAP and GlxR to determine the extent to which key inter- and intramolecular parameters contribute to negative cooperativity in CRP/FNR family transcription factors. We further demonstrate that amino acids that contribute significantly to allosteric control are more likely to be conserved in variant proteins from diverse species. The theoretical and experimental work and associated data analysis provide both a significant advance in our understanding of the mechanisms that underpin the dynamic regulation of allostery and also a means for informed rational engineering of cooperativity in proteins.

## Results

### An ENM for CAP Correctly Predicts Negative Allostery

To computationally address cases of allostery that arise from fluctuation-modification, without conformational change, requires a very different approach from those corresponding to the classic Monod-Wyman-Changeaux case of conformational switching. On the one hand, fully atomistic simulations are not capable of attaining, in most cases, the long dynamical time scales explored by the slow, global dynamic modes whose thermodynamics are essential for the effect. On the other hand, because these modes by their nature integrate many local interactions into their effective geometries and potentials, coarser-grained models of protein structure can possibly provide sufficiently accurate calculations of the relevant dynamics, while allowing the computation of dynamics to the necessary timescales. Models that represent protein structures by Cα-atom positions alone reproduce low-frequency modes well in comparison to experimental data [21],[30]. We therefore used the co-ordinates from a high-resolution crystal structure determination of the full-length cAMP bound CAP homodimer to construct an ENM [31] for the apoprotein as well as single and double ligand bound holoprotein states (Figure S1). Free energies, Δ*G*, were calculated using the full harmonic solution, and the negatively cooperative binding of cAMP to wild-type full-length CAP confirmed by calculating a positive value for ΔΔ*G* = (Δ*G*_holo2_−Δ*G*_holo1_)−(Δ*G*_holo1_−Δ*G*_apo_) = 179 cal mol^−1^ consistent with experimentally obtained values (Table S2) [32]–[35]. To confirm that the total motion within the ENM is not an artefact of coarse-graining, we also carried out molecular dynamics simulations [36] with full atomistic detail, including an explicit water model, and performed principle component analysis (PCA) on the generated trajectories [37]. B factors represent the convolution of static and dynamic disorder in the crystal. Dynamic disorder can be attributed to local motions of individual atoms, whereas static disorder represents different atomic positions in the individual protein molecules. The experimental B factors, albeit constrained by crystal packing, therefore represent a reasonable approximation of the local motions in solution [38]. ENMs and atomistic PCAs represent overall unconstrained dynamic motions and hence show much larger deviations in the termini and the flexible loop regions (for example, residues 150–175 of Figure S2). The crystallographic B factor data were qualitatively well represented at either scale of coarse-graining (Figure S2a) and the distribution of the normal mode frequencies agreed well between ENM and PCA (Figure S2b). The total predicted motion within the ENM, at least at the level of B factors and low-frequency mode structure, is therefore similar to other methods of analysis and not an arbitrary feature of the model. Since the fluctuation-induced allosteric effect arises from the low-frequency structure of the protein dynamics, the ENM level of analysis applies to the experimental phenomena studied here.

We hypothesized that if side-chain replacement on amino acids at sites distinct from the cAMP binding site of CAP do not cause conformational rearrangement, yet increase or decrease amino acid side chain hydrophobic or electrostatic forces in their local environments, the normal modes of protein motion would be altered without significant structural changes. If these changes to the normal modes have sufficiently global effects, they will in turn modify cooperativity between the cAMP binding sites through an entropic contribution to the binding free energy. Amino acid side chain replacement can therefore act as a sensitive probe of the contribution of side chain connectivity to cooperativity and the underlying mechanism for allostery within the elastic structure of the protein. The change in allosteric free energy (ΔΔ*G*) as a function of altering the entire primary amino acid sequence (one residue at a time) can therefore be viewed as a quantitative map of the contribution of the normal modes to cooperativity. Such a quantitative map can be constructed either by simulation or experiment; in practice, it is convenient, as we demonstrate below, to use simulation of the entire allosteric map to guide mutagenesis for experimental study. We therefore performed a scanning computational mutagenesis of the entire CAP protein to investigate the influence of side chain connectivity on cooperativity via their influence on the normal modes.

**Figure 1:**
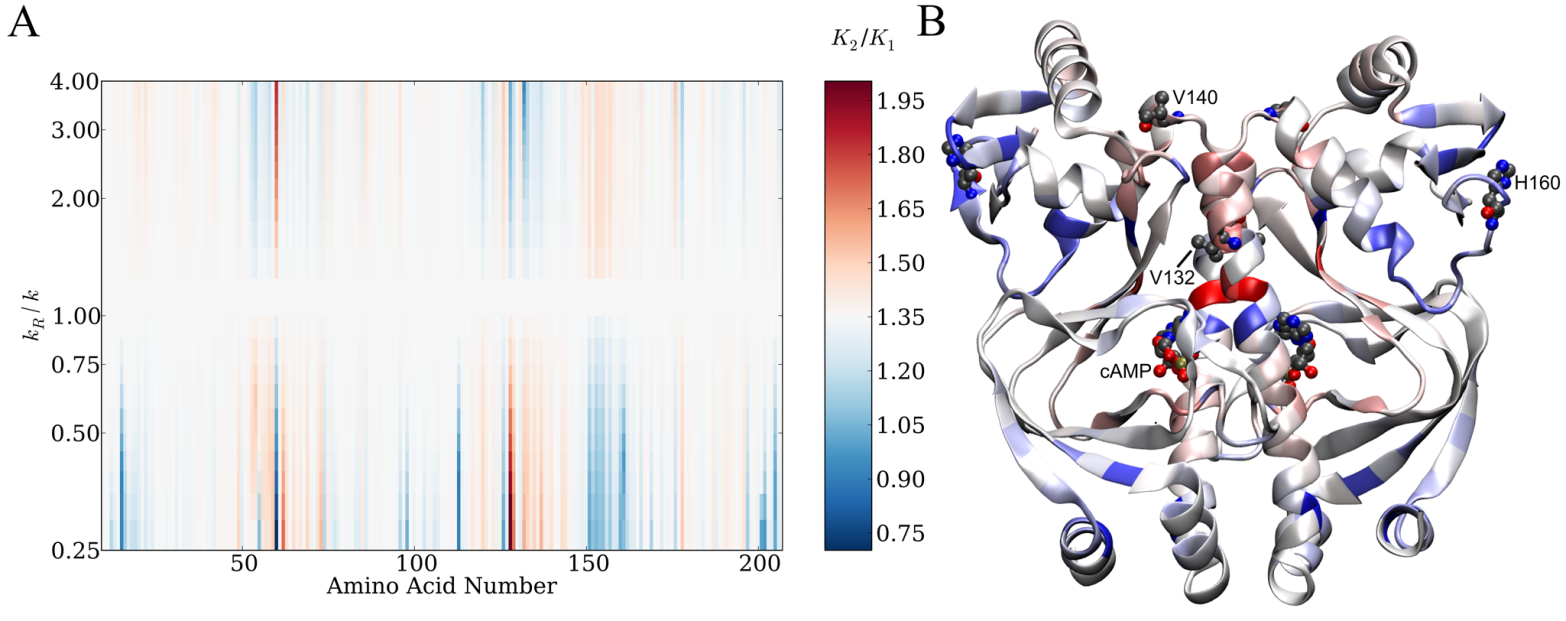
A global map for dynamic regulation of allostery in CAP. (A) Global map for the ENM plotting amino acid number for the CAP monomer and dimensionless change in spring constant (*k*_R_/*k*; corresponds to *k*_amino acid number_/relative spring strength). The colour chart represents changes in the ratio of the second to first dissociation constants for cAMP. White corresponds to values of *K*_2_/*K*_1_ predicted by the wild-type ENM. Red corresponds to increased values of *K*_2_/*K*_1_ (increased negative cooperativity) and blue corresponds to decreased values of *K*_2_/*K*_1_ (decreased negative cooperativity and positive cooperativity). (B) The global map plotted in real space onto the wild-type CAP homodimer structure at *k*_R_/*k* = 0.25. The specific residues investigated in this study are indicated.

Changing the effective elastic potential between protein backbone carbon atoms in the neighbourhood of each residue of the ENM in turn and calculating effects on ΔΔ*G* was used to determine the scanning computational mutagenesis map. The increase and decrease in elastic potential in the ENM was hypothesized to simulate the effects of local strengthening and weakening of side chain interactions in CAP. A color-coded map corresponding to altered cooperativity with changing local interaction strength is plotted graphically by amino acid residue (Figure 1a) and in real space (Figure 1b). The global map for the ENM (Figure 1a) demonstrates large regions where cooperativity is susceptible to control by altering side chain connectivity. It is important to note that these control regions are not necessarily adjacent to the cAMP-binding site. For example, regions corresponding to amino acids 127–137 (at the interface between the two monomers) and 150–162 (within the DNA binding domain, far from both the dimer interface and cAMP binding regions) appear to exercise considerable control over cooperativity without contributing to a spatially distinct dynamic pathway and without direct interference with the cAMP binding site.

### Residues of CAP That Modify the Normal Modes Predictably Alter Allostery

**Figure 2:**
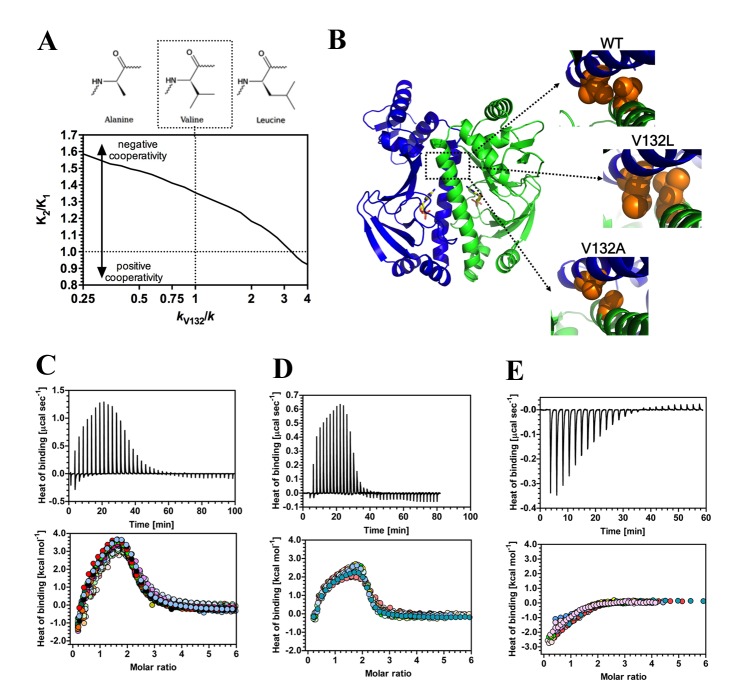
The influence of third-site mutations on allostery in CAP. (A) Predicted influence of mutation of V132 on allostery in CAP. The chart represents the ratio of the second to first dissociation constants for cAMP (*K*_2_/*K*_1_) plotted against spring constant at V132 (*k*_V132_/*k*). The structures are the proposed corresponding mutations. (B) Close-up of the X-ray crystal structures for CAP variants showing the hydrophobic interaction surface at amino acid 132 in wild-type, V132L, and V132A proteins. (C–E) ITC traces (upper panel) and binding isotherms (lower panel; the different coloured symbols represent individual experiments) for the calorimetric titration of cAMP to CAP wild-type (C), V132L (D), or V132A (E). The thermodynamic parameters obtained are summarized in Tables 1 and S2.

To experimentally test the model and demonstrate rational engineering and control of allostery, we selected the residues of CAP highlighted in Figure 1b. We examined amino acids predicted to show altered (V132, H160) or neutral (V140) responses to altered amino acid side chain interactions (Table 1). The removal (V132A) or addition (V132L) of a side chain methyl group of V132 was engineered to decrease and increase, respectively, the strength of hydrophobic interaction across the dimer interface. Computation predicted that these mutations would result in more negative and positive cooperativity in CAP, respectively (Figure 2a) and that the most important contacts contributing to this effect were with L62 and V132 of the opposing monomer (Figure S3b). High-resolution X-ray crystal structures of CAP mutants V132A and V132L demonstrated that these variants possessed decreased and increased hydrophobic interactions across the dimer interface, respectively (Figure 2b). Comparison of variant crystal structures with wild-type demonstrated that there was no statistically significant change in structure (Figure S4, Table S1). Cooperativity for cAMP binding was studied by isothermal titration calorimetry (ITC) for wild-type, V132A, and V132L proteins to examine whether the experimentally observed changes in cooperativity matched computational predictions (Figure 2c–e, Table 1). The ITC data were well-described by a three-site model, with two major and one minor cAMP binding site (Figure S5) [39] and allowed derivation of the thermodynamic parameters for all proteins (Table S2). The qualitative computational prediction for altered cAMP cooperativity was tested experimentally including a significant controlled inversion of the sign of the cAMP cooperativity (V132L). The thermodynamic parameters for wild-type CAP demonstrated an overall favourable entropy change and unfavourable enthalpy change on binding the second molecule of cAMP consistent with a previous report [39]. A previous study of the truncated CAP ligand-binding domain demonstrated that binding of the second molecule of cAMP was entropically unfavoured [10]. The difference in thermodynamics between our experiments (Table S2) and previous experiments using the ligand-binding domain alone [10] is therefore likely due to the contribution of motions of the DNA binding domain [40]. This interpretation is supported by previous analysis that has calculated the thermodynamic contribution of the DNA binding domains in the switch to the active conformation [41]. Previous calculations and experiments anticipate that, while the contribution of the normal modes to allostery is entropically controlled (in terms of the net allosteric free energy), coupling of the low-frequency modes to side-chain motion generically gives rise to additional, but compensating, contributions to enthalpy and entropy and this is observed in our thermodynamic data (Table S2) [9]. It is notable that, due to this self-cancelling of the contribution of local fast modes within the total free energy, the entropically driven ENM is able to predict qualitative changes to experimental cooperativity despite the local mode contribution of enthalpy to overall thermodynamics.

**Table 1:** Calculated and experimental cAMP affinities for CAP proteins.

CAP Protein	*K*_2_/*K*_1_ (ENM)	S.E.M. (*n*)	*p* Value	Mean *K*_2_/*K*_1_ (ITC)	S.E.M. (*n*)	*p* Value
Wild-type	1.35	0.01 (16)	—	1.68	0.04 (32)	—
V132A *k*_V132_/*k* = 0.25	1.59	0.01 (16)	<0.001	4.78	0.33 (20)	<0.001
V132L *k*_V132_/*k* = 4	0.91	0.02 (16)	<0.001	0.58	0.03 (17)	<0.001
H160L *k*_H160_/*k* = 0.25	1.05	0.02 (16)	<0.001	1.36	0.03 (31)	<0.001
V140A *k*_V140_/*k* = 0.25, *k*_C179_/*k* = 4	1.02	0.01 (16)	<0.001	0.61	0.05 (29)	<0.001
V140L, *k*_V140_/*k* = 4	1.31	0.01 (16)	0.733	1.56	0.05 (27)	0.999

The ratio of the second to first dissociation constants for cAMP (*K*_2_/*K*_1_) for wild-type and mutant CAP proteins were calculated from the ENMs or obtained by ITC. The *p* value is for a comparison of means to the wild-type.

**Figure 3:**
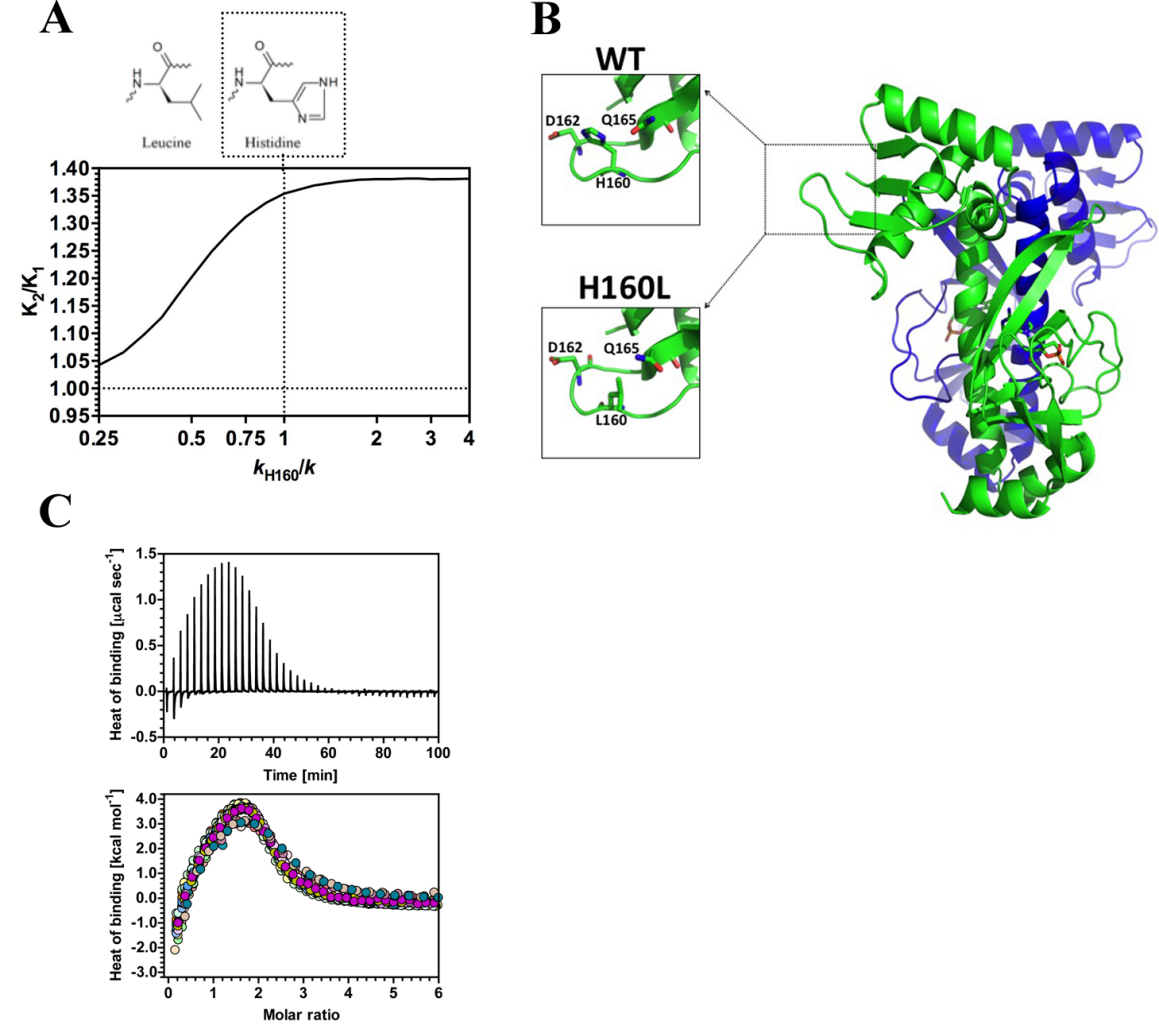
The influence of third-site mutations on allostery in CAP. (A) Predicted influence of mutation of H160 on allostery in CAP. The chart represents the ratio of the second to first dissociation constants for cAMP (*K*_2_/*K*_1_) plotted against spring constant at H160 (*k*_H160_/*k*). The structure is the proposed corresponding mutation. (B) X-ray crystal structures for CAP showing the hydrogen bonding network at amino 160 in wild-type and H160L proteins. (C) ITC trace (upper panel) and binding isotherm (lower panel; the different coloured symbols represent individual experiments) for the calorimetric titration of cAMP to CAP H160L. The thermodynamic parameters obtained are shown in Tables 1 and S2.

The ENM calculations predicted a reduction in the negative cooperativity of CAP in response to a reduction in the strength of the local interactions of residue H160 (Figure 3a). In particular, H160 was predicted to form interactions that contribute to allostery with D162 and Q165 (Figure S3a). The mutation H160L was predicted to break these interactions while maintaining side chain bulk; this was confirmed by X-ray crystallography of the H160L CAP protein (Figure 3b). No overall change in H160L protein structure was evident compared to wild-type (Figure S4, Table S1). ITC experiments (Figure 3c) demonstrated that cooperativity for cAMP became less negative as predicted by computation (Table 1). This crucial experiment demonstrates that altering low-frequency motions at a site distant from both the ligand binding site as well as the dimer interface, and from any presumed physical pathway of structural change connecting these sites, can nonetheless give predictable effects on cooperativity.

**Figure 4:**
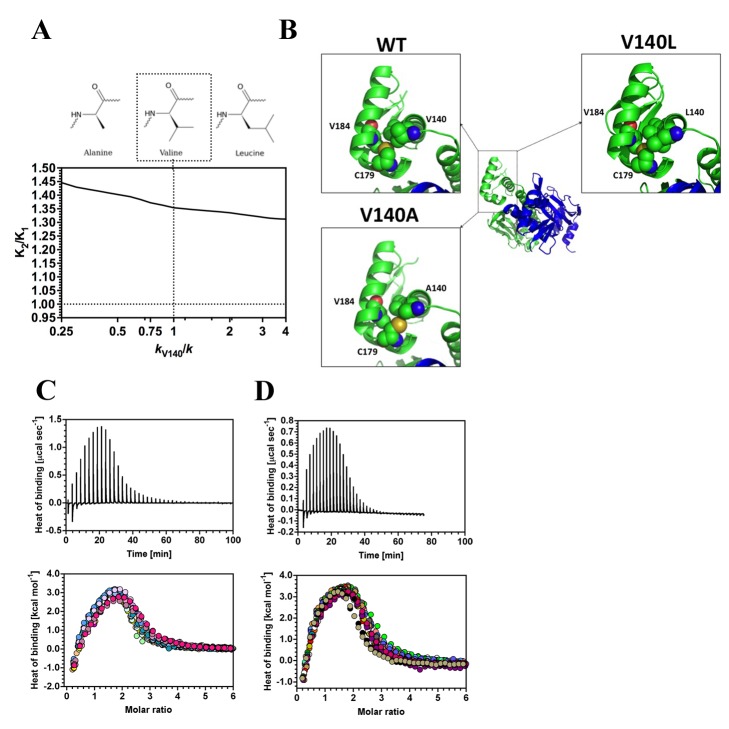
The influence of third-site mutations on allostery in CAP. (A) Predicted influence of mutation of V140 on allostery in CAP. The chart represents the ratio of the second to first dissociation constants for cAMP (*K*_2_/*K*_1_) plotted against spring constant at V140 (*k*_V140_/*k*). The structures are the proposed corresponding mutations. (B) X-ray crystal structures for CAP showing the hydrophobic interactions at amino 140 in wild-type, V140L, and V140A proteins. (C–D) ITC traces (upper panel) and binding isotherms (lower panel; the different coloured symbols represent individual experiments) for the calorimetric titration of cAMP to CAP V140L (C) and V140A (D) proteins. The thermodynamic parameters obtained are shown in Tables 1 and S2.

Altering local interactions associated with V140 was predicted by the ENM to have minimal effects on cooperativity (Figure 4a) despite significant local hydrophobic interactions; we therefore examined the effect of decreased and increased local hydrophobic interactions in V140A and V140L variants as a control experiment. The V140L mutant protein had no discernible effect on protein structure (Figure S4). As predicted by the ENM mutagenesis, measurement of cooperativity for cAMP in V140L by ITC (Figure 4c) showed no differences when compared to wild-type (Table 1). Interestingly, although V140A protein showed no global change in structure (Figure S4), there is, in this mutation, a significant local conformational change evident in the crystal structure where the mutated V140A residue formed a new hydrophobic contact with the rotated side chain of C179 that is not present in the wild-type or V140L proteins (Figure 4b). When included in the model, simulated as *k*_C179_/*k* = 4, this new contact revealed new interactions within the monomer (Figure S3a) that drove CAP towards positive cooperativity on simulation (Table 1). ITC experiments (Figure 4d) demonstrated that this CAP variant with the identified side chain rearrangement was positively cooperative, thus supporting the qualitative prediction of the model.

A bar graph for the calculated and observed values for *K*_2_/*K*_1_ revealed the agreement in the direction of the change of cooperativity on simulation and experiment (Figure S6a). A plot of the experimentally observed value for *K*_2_/*K*_1_ against that predicted from the ENM demonstrated a correlated relationship where observed increases to *K*_2_/*K*_1_ are associated with similar changes to *K*_2_/*K*_1_ by the ENM (Figure S6b). The consistency in prediction by the ENM and the quantitative correlation between predicted and observed changes do not support the notion that the agreement between experiment and the ENM is due to a chance occurrence.

**Figure 5:**
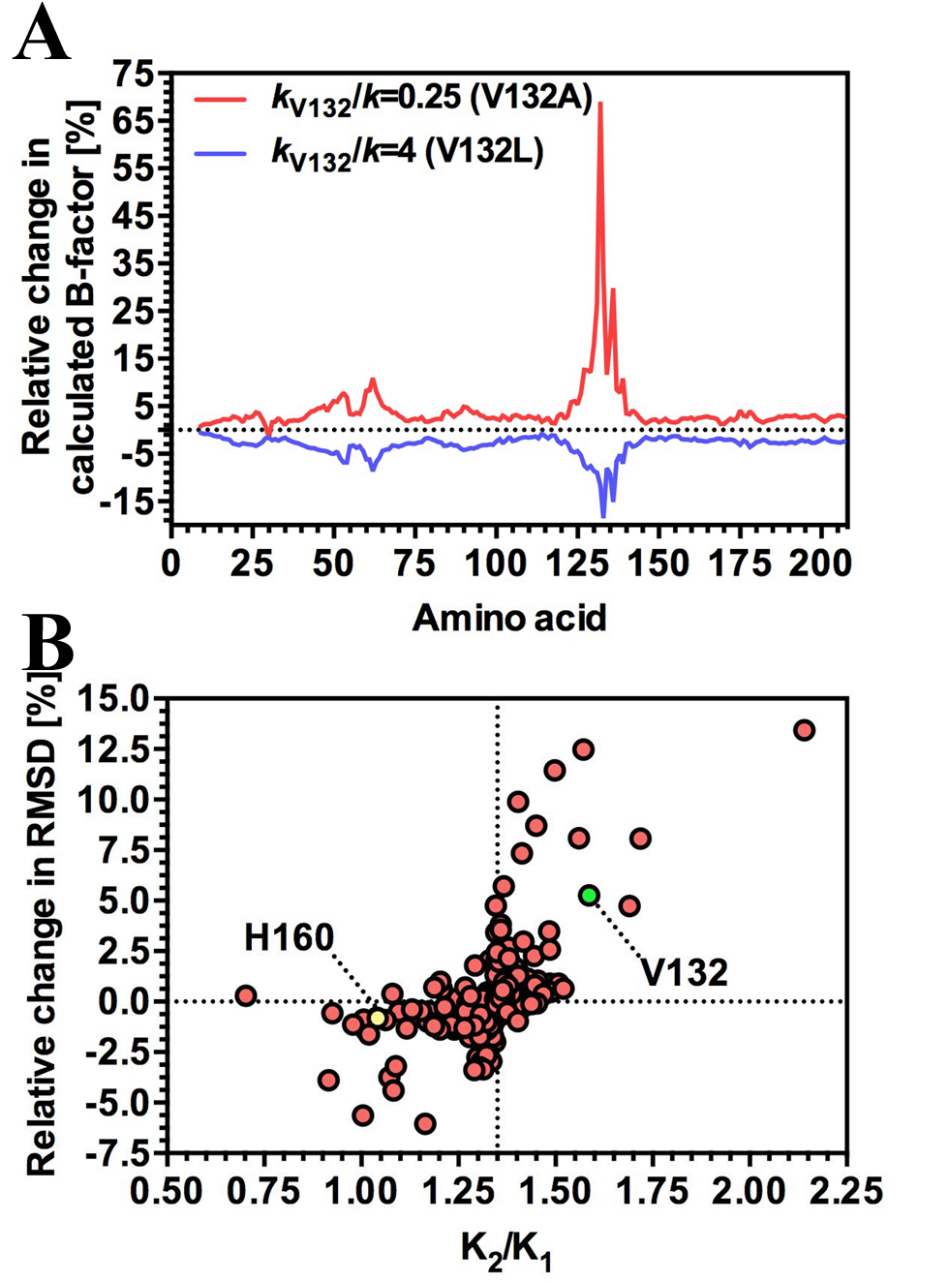
Mapping local dynamics in CAP. (A) The effect of mutation of V132 on local dynamics over the CAP monomer. The chart represents the percentage variation in the calculated B-factor from the wild-type ENM plotted against amino acid number. (B) The relationship between local dynamics at the cAMP binding site and cooperativity. The chart represents the percentage variation in the root mean square deviation of the simulated CAP variant Cα atom from the wild-type crystal structure at the cAMP binding site (amino acids 71–74, 83–85, and 121) plotted against the ratio of the second to first dissociation constants for cAMP (*K*_2_/*K*_1_) at *k*_R_/*k* = 0.25. The positions of simulated mutations at V132 and H160 are indicated.

The ENM can provide further insight into the mechanism by which allosteric control is associated with alterations to the normal modes. No global structural changes were induced in the ENM simulations or were evident from crystal structures of variant proteins; only the pattern of coupled low-frequency fluctuations was modified by the simulated side-chain mutations. This appearance of “control at a distance” in the CAP homodimer is explained, through contributions to binding entropy, if there are correlations in the low-frequency motions between cAMP binding sites and if ligand binding or side chain mutation modifies this correlation [42]. As all fluctuating systems dominated by locally harmonic interactions possess a structure of normal modes, with just such distant correlations, they suggest the mechanism for allostery in CAP. To examine whether the mutations studied here can have such distant effects, we calculated the change to local Cα flexibility in the case of tightening and loosening side chain interactions at V132 at the dimer interface (Figure 5a). Modifications to simulated backbone flexibility are present throughout CAP with varying amplitude and furthermore follow opposite signs at *k*_V132_/*k* = 0.25 (V132A) and *k*_V132_/*k* = 4 (V132L). For example, *k*_V132_/*k* = 4 shows significant tightening of the protein (compare Figure 5a and Figure S3b). An examination of the effect of simulated mutations at V140 and H160 on nonlocal Cα flexibility reinforces this finding (Figure S7). The predominantly neutral mutation, V140L, simulated as *k*_V140_/*k* = 4 has little effect on protein backbone flexibility, except at sites where V140 has calculated interactions, consistent with the absence of any effect on allostery on both simulation and experiment. In the case of H160 (*k*_H160_/*k* = 0.25; at a surface loop distant from both the cAMP binding site and dimer interface) and V140A (*k*_C179_/*k* = 4, *k*_V140_/*k* = 0.25), the simulated mutations create a uniform decrease in flexibility throughout the monomer except for the straightforward loosening/tightening at the site of the mutations. There is a general trend, therefore, for those simulated mutations that decrease negative cooperativity to be associated with decreased protein backbone motion nonlocally.

A specific requirement of global low-frequency motion as an underpinning mechanism for allostery at a distance is a coupling between protein motion and the behaviour of the cAMP-binding site. We find that the loosening and tightening effects of simulated mutations is correlated with significant modulation of backbone flexibility in the region of the cAMP-binding site (amino acids 71–74, 83–85, and 121) (Figure 5b). The figure shows that, in general, changes in root-mean-square deviation (rmsd) at the ligand-binding site induced by mutation correlate (in this case, *k*_R_/*k* = 0.25) with cooperativity. Mutations that increase motion at the ligand bind site are associated with an increase in the extent of negative cooperativity and vice versa. This is entirely consistent with the controlling entropic allosteric mechanism in these cases, providing that cAMP binding has the effect of increasing local rigidity. This interaction between the heightened local motions following the first cAMP-binding event creates an entropic contribution to negative cooperativity in ΔΔ*G* [9]. Heightened fluctuation at the second binding site (on binding the first molecule of cAMP) is a general mechanism for negative cooperativity without conformational change [6]. Positive cooperativity without conformational change can be induced by reducing the fluctuation amplitude (for example, the MetJ transcription factor of *E. coli* [9]).

### Global Low-Frequency Dynamics Regulates Allostery in the CRP/FNR Family Transcription Factor GlxR

**Figure 6:**
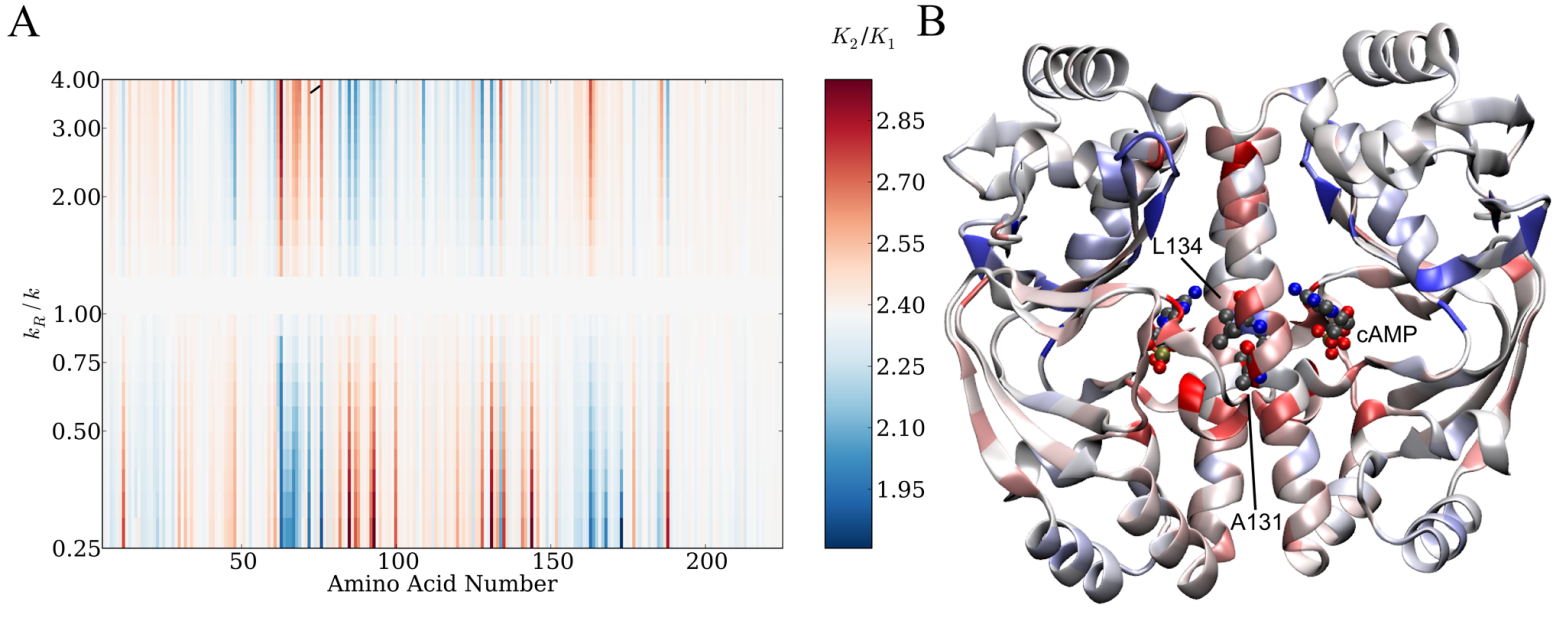
A global map for dynamic regulation of allostery in GlxR. (A) Global map for the ENM plotting amino acid number for the GlxR monomer and dimensionless change in spring constant (*k*_R_/*k*). (B) The global map plotted in real space onto the wild-type GlxR homodimer structure at *k*_R_/*k* = 0.25. The specific residues investigated in this study are indicated.

**Figure 7:**
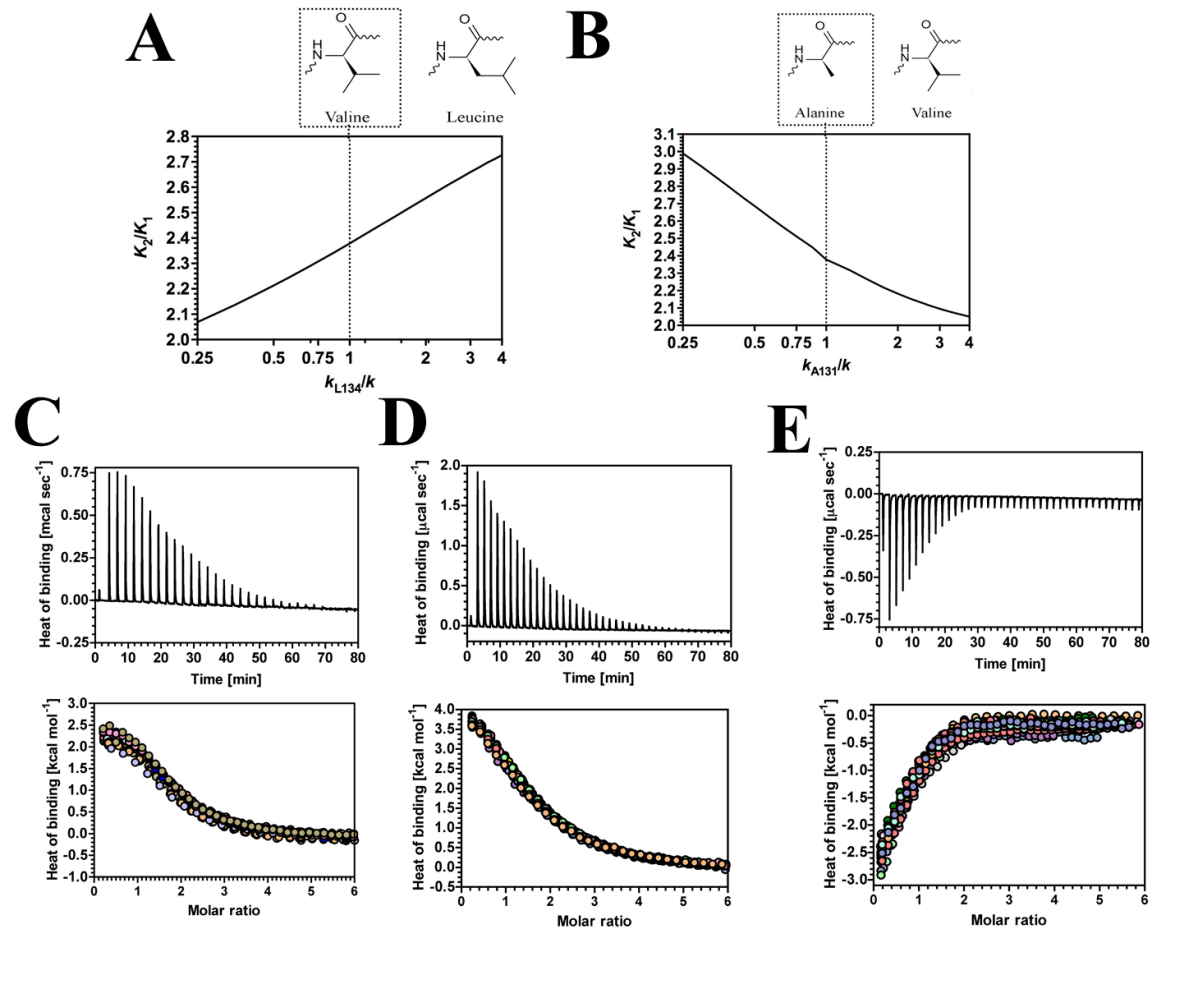
The influence of third-site mutations on allostery in GlxR. (A) Predicted influence of mutation of L134 on allostery in GlxR. The chart represents the ratio of the second to first dissociation constants for cAMP (*K*_2_/*K*_1_) plotted against spring constant at L134 (*k*_L134_/*k*). The structures are the proposed corresponding mutations. (B) Predicted influence of mutation of A131 on allostery in GlxR. The chart represents the ratio of the second to first dissociation constants for cAMP (*K*_2_/*K*_1_) plotted against spring constant at A131 (*k*_A131_/*k*). The structures are the proposed corresponding mutations. (C–E) ITC traces (upper panel) and binding isotherms (lower panel; the different coloured symbols represent individual experiments) for the calorimetric titration of cAMP to GlxR wild-type (C), L131V (D), and A131V (D) proteins. The thermodynamic parameters obtained are shown in Tables 2 and S3.

Studies using CAP have successfully demonstrated that changes to global low-frequency protein dynamics are associated with allostery. We investigated another protein to explore the more general applicability of the mechanism. GlxR of *C. glutamicum* is a cAMP binding homodimeric transcription factor of the CRP/FNR family that activates genes required for aerobic respiration, glycolysis, and ATP synthesis [43],[44]. We solved the X-ray crystal structure of the GlxR apoprotein to produce an ENM for the non-cAMP bound state [45]. Coordinates from an available crystal structure determination of the full-length cAMP bound GlxR homodimer allowed us to construct an ENM for the single and double ligand bound holoprotein states. Examination of the structures for GlxR in the apo and holo forms revealed no significant difference in structure. GlxR therefore represents a new exemplar for allostery in the absence of conformation change. Free energies, calculated from ENMs for GlxR, predicted considerably greater negative cooperative binding of cAMP (*K*_2_/*K*_1_ = 2.37; ΔΔ*G* = 513 cal mol^−1^) than for CAP (*K*_2_/*K*_1_ = 1.35; ΔΔ*G* = 179 cal mol^−1^). This prediction of enhanced negative cooperativity was confirmed on experiment with an observed value for *K*_2_/*K*_1_ of 19.47 (Table 2). A computational scanning mutagenesis map was produced for GlxR, as done previously for CAP, and altered cooperativity with changing local interaction strength is plotted graphically by amino acid residue (Figure 6a) and in real space (Figure 6b). Both local tightening and loosening across the dimer interface, depending on the residue, was predicted to reduce negative cooperativity and therefore provides a robust experimental test of the model. We generated dimer interface loosening (*k*_L134_/*k* = 0.25; L134V; Figure 7a) and tightening (*k*_A131_/*k* = 4; A131V; Figure 7b) GlxR variants and compared simulated and experimental values for cooperativity in these proteins. Both L134V and A131V showed a clear reduction in negative cooperativity, as predicted, when compared to wild-type (Table 2) by ITC (Figure 7c–e), despite the fact that the mutants have opposing effects on hydrophobic interactions across the dimer interface. Allostery is therefore correlated with global low-frequency dynamics in an additional CRP/FNR family transcription factor.

**Table 2:** Calculated and experimental cAMP affinities for GlxR proteins.

GlxR Protein	*K*_2_/*K*_1_ (ENM)	Mean *K*_2_/*K*_1_ (ITC)	S.E.M. (*n*)	*p* Value
Wild-type	2.37	19.47	1.12 (18)	-
L134V *k*_L134_/*k* = 0.25	2.07	4.34	0.21 (26)	<0.001
A131V *k*_A131_/*k* = 4	2.05	4.36	0.21 (30)	<0.001

The ratio of the second to first dissociation constants for cAMP (*K*_2_/*K*_1_) for wild-type and mutant GlxR proteins were calculated from the ENM or obtained by ITC. The *p* value is for a comparison of means to the wild-type.

### Determining Design Parameters for Mapping Dynamically Driven Allostery

**Figure 8:**
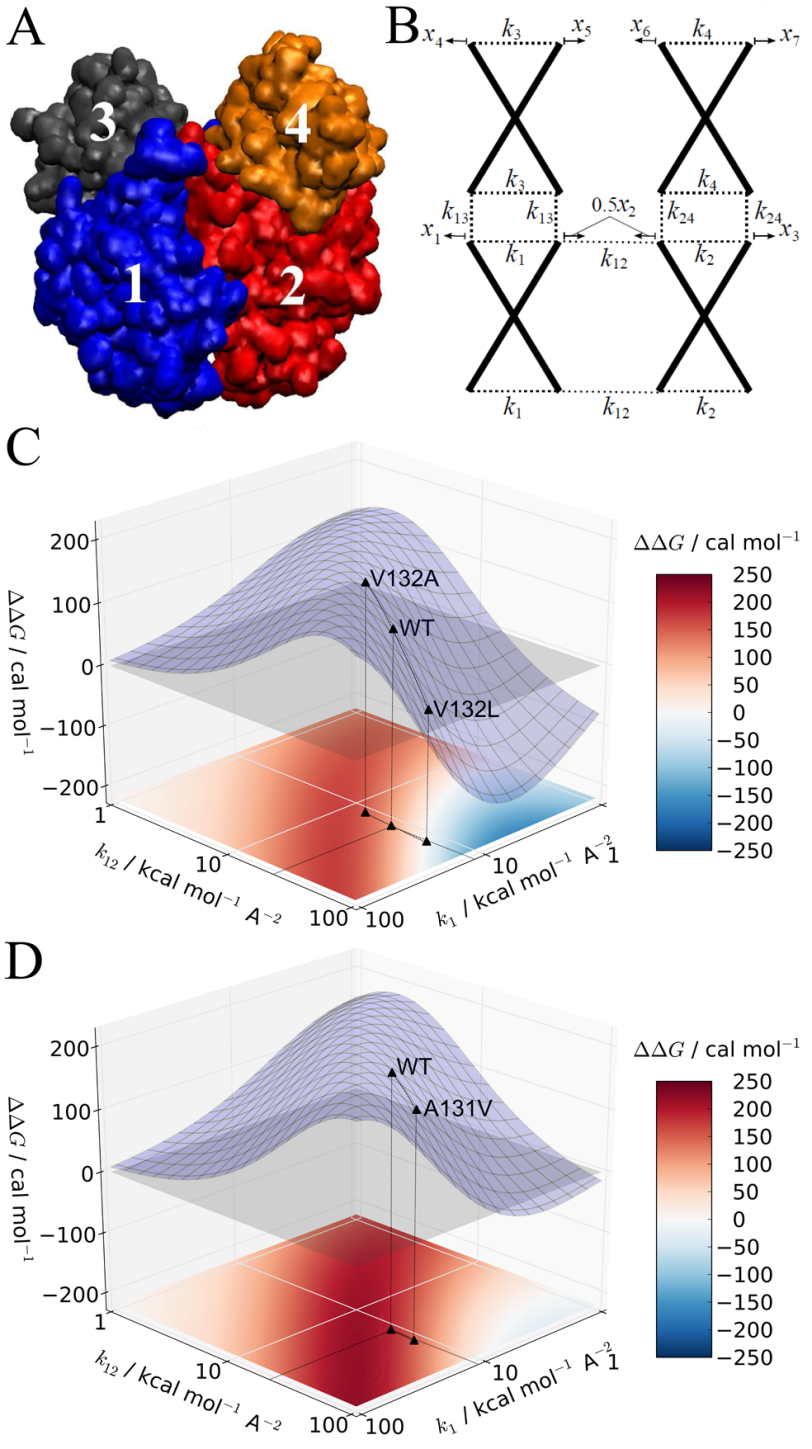
Super-coarse-grained models of CRP/FNR family transcription factors. (A) The elastic block representation that emerges from constraining all residues whose relative spatial fluctuations are less than 3 Å to a single rigid domain. This procedure creates two blocks in each monomer. (B) The corresponding ENM model in which each block is accorded a single internal mode. The plots of cooperativity as a function of the reduced two-dimensional design space of the effective internal and coupling elastic strengths of the super-coarse-grained model are shown for CAP (C) and GlxR (D). All other parameters for the models identified in (B) are set to wild-type values deduced from the full ENMs.

Our findings indicate general biophysical principles that describe the emergence of negative cooperativity in CRP/FNR family transcription factors through the allosteric modulation of normal modes. The property that allosteric effects are carried in general by the more globally distributed, and so typically longer wavelength, normal modes motivated the exploration of the underlying physics by coarse-graining the CAP and GlxR representations even further into rotational-translational block representations [46]. Two coarse-grained blocks per monomer (one is the entire DNA-binding region, coupled only to the other block of its own monomer) emerged naturally from the many residue–residue couplings internal to and between monomers at the molecular level. Figure 8a and 8b display the block structure and the corresponding “super-coarse-grained” model. A single representative internal mode within each dynamically tight block and the coupling strengths between the blocks (including coupling across the dimer interface) were investigated as “design parameters” for a general class of cooperative homodimer. Figure 8c (CAP) and 8d (GlxR) show allosteric cooperativity, calculated at this high level of coarse-graining, as a function of the integrated coupling strengths within the ligand binding domain (*k*_1_) and between monomers (*k*_12_). Points below and above the *z* = 0 plane correspond to positive and negative cooperativity, respectively. The wild-type proteins for both CAP and GlxR are offset from the maxima of anti-cooperative ridges in the two-dimensional free energy landscapes that emerge. At this position, loosening coupling internal to monomers (*k*_1_) moves the system into a basin of less negative cooperativity (GlxR) or positive cooperativity (CAP), while loosening in the coupling region (*k*_12_) moves the system for both CAP and GlxR to the top of the ridge (red) to increase negative cooperativity. Further analysis demonstrated consistency in the negative cooperativity arising through the normal modes in the ENM and in the super-coarse-grained model. For example, the simulated loosening (*k*_V132_/*k* = 0.25; V132A) and tightening (*k*_V132_/*k* = 4; V132L) mutations of the CAP ENM and the tightening (*k*_A131_/*k* = 4; A131V) mutation of GlxR alter cooperativity through generating effective changes in *k*_12_ at the super-coarse-grained level. The super-coarse-grained model therefore effectively reveals the critical intra- and intermolecular parameters that associate with cooperativity and how these parameters can be altered to move within the allosteric free energy landscape.

### Amino Acid Residues That Contribute to Allostery in CAP Are Evolutionarily Conserved

**Figure 9:**
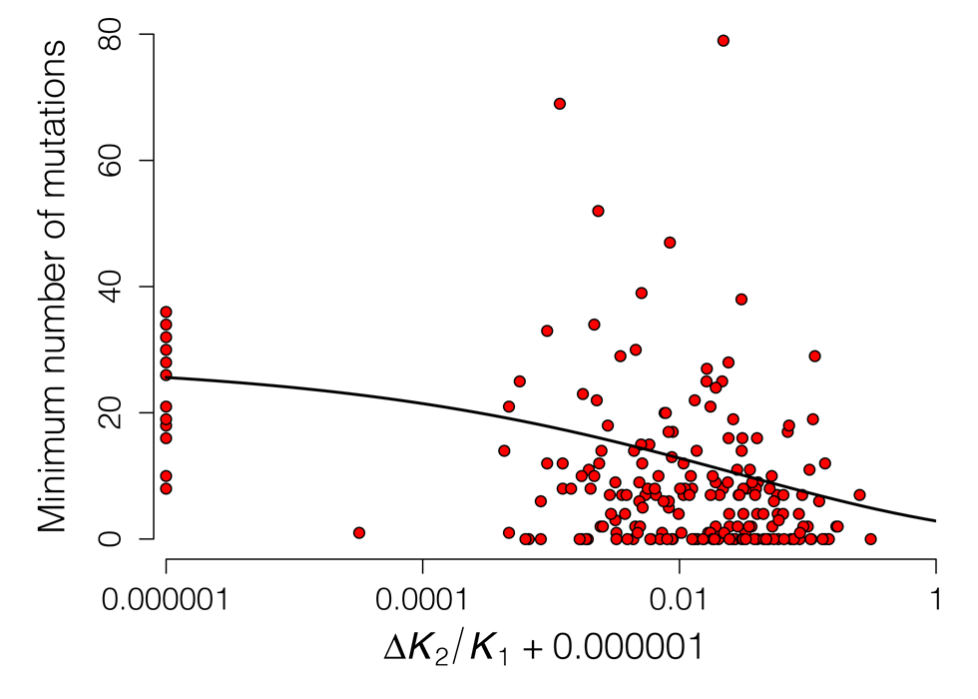
CAP residues that contribute to allostery are conserved in variants from diverse bacterial species. Relation between the minimum number of times an amino acid needs to exhibit a mutation to explain the variation in the set of sequence data ( = 165 proteins) and the contribution of that amino acid to allostery (Δ*K*_2_/*K*_1_ for that amino acid at *k*_R_/*k* = 0.25). Each circle corresponds to one of 210 amino acids. The best fitting model predicts that mean mutation rate declines with allostery (solid line). Note that absolute allosteric values have been used and increased by 0.000001 to allow plotting of zeros.

If cooperativity confers a selective advantage on the organism, then the allosteric free energy landscape can also be viewed as evolutionary landscape. In this case, the position of a protein within the landscape depends upon selection pressures that impact upon *k*_1_ and *k*_12_. This general hypothesis can be used to make an additional significant experimental prediction. If the similar position of CAP and GlxR within their respective free energy landscapes is the result of a selection pressure, then we predict that amino acids that contribute significantly to quantitative allosteric control (Figure 1a and 6d) will be more invariant in related proteins from different species. We therefore examined 163 CAP variants from diverse bacterial species and plotted the frequency of mutation of each amino acid residue against the contribution of that amino acid to allostery (defined as absolute change (Δ) in *K*_2_/*K*_1_ for that amino acid in the canonical CAP ENM at *k*_R_/*k* = 0.25). We found evidence that the rate at which an amino acid mutates is negatively related to Δ*K*_2_/*K*_1_ (LRT, *G*_2_ = 33.7, *p*<0.001; Figure 9). The coefficient quantifying this decrease, *β*_1_, was significantly different from zero [95% CI = (−3.34,−1.49)]. Amino acids of CAP that contribute to allostery through regulation of low-frequency protein dynamics are therefore more likely to be conserved in CAP variants through their contribution to protein function. Note that a test for overdispersion was significant, even after allostery had been accounted for (LRT, *G*_1_ = 1,663.9, *p*<0.001), suggesting that other variables also have an influence on mutation rates.

## Discussion

Here we demonstrate that negative allostery in CRP/FNR family transcription factors is correlated with modulation of the normal modes of protein motion on ligand binding in the absence of conformational change. The model makes key predictions that we test at select sites of the CAP and GlxR proteins, the latter identified as an important new exemplar for allostery in the absence of conformation change. The alterations in protein flexibility that are a signature for allostery in CRP/FNR family transcription factors are a consequence of the global nature of those normal modes responsible and mutations that predictably alter cooperativity do so by influencing protein backbone flexibility. Our theory describes how allostery can arise from changes to low-frequency dynamics in the absence of any mean structural change. The theory is particularly significant as it describes allostery as a natural consequence of the dynamic properties of a protein without a requirement for spatially localised dynamic pathways between allosteric sites. The allostery observed is unlikely to have microheterogeneity as an alternative explanation as all CAP proteins crystallised as a single superimposable structure. Any form of heterogeneity reduces the likelihood of forming ordered crystals [47]. Microheterogeneity is therefore not supported as a molecular cause for allostery in CAP.

The possibility of a direct interaction between cAMP binding sites might also be considered as a mechanism to explain the allostery observed. The closest distance between the two cAMP molecules in the CAP dimer is 9.5 Å (the distance between the N6 atoms of the adenine ring). Although it is impossible to conclusively eliminate small local changes that binding of the first molecule of cAMP has at the second site, no conformational changes have been reported in this region in previous NMR studies, making this explanation unlikely. The possibility of a direct interaction is made even more unlikely as, similar as to that described above, any invoked direct interaction between cAMP binding sites would have to consistently match not only the qualitative aspects of the computational predictions for the role of the global modes, but also their quantitative correlation with the observed experimental values. Analysis of the relationship between Cartesian distance and protein motions demonstrated strongly correlated motions between allosteric sites at distances of <10–20 Å [48] and the global normal modes are a suitable candidate to mediate such correlations in CRP/FNR family transcription factors.

The range of available sites for side chain mutagenesis of CRP/FNR family transcription factors do not constitute as large a set of separate and independent control parameters as at first seems, but in a good approximation explore a lower dimensional space (i.e., reducing the very high dimensional parameter-space of the entire number of residues, just one slice of which is represented in Figures 1a and 6d, to the two-dimensional parameter spaces of Figure 8c–d). We hypothesize that this two-dimensional parameter space is, in turn, related to an evolutionary landscape for a protein. In the case of CAP and GlxR, our analysis reveals that evolutionary selection has resulted in the location of the proteins in a region close to maximizing negative cooperativity. The extent of negative cooperativity in CAP is generally small (ΔΔ*G* = 0.3 kcal mol^−1^). However, the scale of biologically relevant cooperative effects is set by the thermal energy (*RT*≈0.6 kcal mol^−1^). The values of ΔΔ*G* observed and manipulated experimentally are those that modulate the concentration range of cAMP to which the system is sensitive by an order of 1. Engineering of cooperativity is therefore possible by manipulating ΔΔ*G*, as described here, with the caveat that it is likely only possible over a thermodynamic range to which the protein is responsive.

We find that there is a selection pressure against mutation of residues that contribute to allostery in CAP variants. A significant question that arises, therefore, is that of the selective advantage provided through negative cooperativity in CAP. In general, the advantages conferred by negative cooperativity in biological systems are not well resolved [49]. It is proposed that negative cooperativity reduces the sensitivity of a system and extends the concentration range over which a response can be observed [50]. In metabolism, recent modelling suggests that there is a significant overall advantage for metabolic pathway flux with components showing negative cooperativity [51],[52]. In transcriptional regulation, negative cooperativity in the binding of D-camphor to the CamR repressor of *Pseudomonas putida* is proposed to enable coupling of high specificity for D-camphor with a physiological response to high concentrations of the metabolite [53]. Against this framework, it is reasonable to conjecture that negative cooperativity in CAP offers a selective advantage by increasing the concentration range over which a transcriptional response can be generated [54]. The decreased sensitivity of the response to cAMP in negative cooperativity might result in a selective advantage through resource conservation when compared to amplifying effect of a signal response in positive cooperativity [50]. The position within the effective parameter space can also allow CAP variants to further tune cooperativity in either direction without a potentially disastrous influence on protein structure and therefore function. Future experiments to experimentally validate the selective advantage provided by negative cooperativity will therefore be crucial and might typically combine high throughput sequencing of extensive mutational libraries of CAP, after selection in *E. coli*, with the simulated mutational map of this study [55].

The super-coarse-graining and finer-grained tools we have developed and tested in this work suggest a route to artificial protein design through modification of protein low-frequency fluctuations without compromise of structure. The mechanism also reflects an important balance between phenomena at different length scales within molecular biology. The role of the global normal modes in conveying allosteric signals requires a similarly coarse-grained picture of the protein to identify and discuss the mechanism. On the other hand, the exquisite specificity to local biochemistry is preserved in the mechanism; a set of single residues, themselves spatially distant from either binding site, exercise significant control on the size (and sign) of the underlying allosteric signal. The delicate interactions of effects at different length scales are missed without such a multiscale approach to the physics of protein dynamics. Changes to the normal modes are presented as an important new theory to describe how allostery can arise in the absence of structural change and provide an important theoretical context within which to frame global issues of allostery in proteins.

## Materials and Methods

### Protein Preparation

The open reading frame corresponding to the full-length CAP protein was cloned into the *Bam*HI and *Hin*dIII sites of pQE30 and mutant variants constructed by site-directed mutagenesis. Wild-type and mutant recombinant protein was expressed from *E. coli* M182 ΔCAP *F^−^* Δ(*lacIPOZY*)X74 *galE*15 *galK*16 *rpsL thi^+^ lambda^−^* [pREP4] for 2 h at 37°C with 1 mM IPTG. Protein was purified using sequential nickel-chelated sepharose affinity and Superdex 75 16/60 size exclusion columns (GE Healthcare). Protein concentration was calculated using the Beer-Lambert Law and a molar extinction coefficient of 20,065 M^−1^ cm^−1^ at 280 nm. Full-length GlxR protein was expressed and purified as previously described [56].

### ITC

Protein was dialyzed against 100 mM KPO_4_ pH 7.8, 200 mM KCl, 2 mM 1-thioglycerol at 4°C. Protein and buffer were degassed under vacuum and degassed buffer used to dilute cAMP ligand. cAMP concentration was calculated using the Beer-Lambert Law and a molar extinction coefficient of 14,650 M^−1^ cm^−1^ at 259 nm. Data were generated using an iTC200 (MicroCal) by typically 40 sequential 1 µL injections of 4–6 mM cAMP into 202 µL 130–400 µM protein. Data for the first injection was routinely discarded as this is affected by diffusion between the syringe and the protein solution during equilibration prior to data collection.

### Data Fitting for ITC

Ligand binding for cAMP to CAP was described by a sequential three-site model (two major and one minor binding site [39]). The presence of three cAMP binding sites in CAP was further confirmed in the crystal structures from this study (Figure S5). A sequential two-site model described ligand binding for cAMP to GlxR. The free ligand concentration, [L], was calculated for each injection using the bisection method, which allowed calculation of the fraction of the protein in each bound state, *F_i_*:
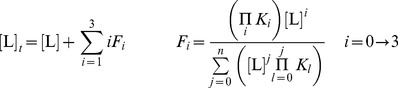
Comparing the calculated heat content, *Q*, to the experimental value allowed calculation of the best fit of the binding constants, *K_i_*, and the binding enthalpies, Δ*H_i_*, using the solver plug-in for Excel:
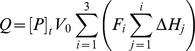



### Statistics

ITC and ENM data for mutant proteins was compared to the wild-type by a comparison of means by one-way ANOVA. Normal distribution of the data was confirmed by the Shapiro-Wilk test. Homogeneity of variances was rejected for ITC data and confirmed for ENM data using the Levene test. ITC data were therefore examined using a Dunnett's T3 post hoc test for pairwise comparisons with unequal variances and ENM data examined using a two-sided Dunnett's post hoc test for pairwise comparisons with equal variances.

### Crystal Structure Determinations

CAP crystals were produced at pH 6.5 with 7–10% (w/v) polyethylene glycol 3350 and 15–20% (v/v) 2-methyl-2,4-pentanediol with 2 mM cAMP in 24-well hanging-drop vapour diffusion plates. Crystals were cryoprotected using mother liquor containing 30% (v/v) glycerol and flash cooled in liquid nitrogen [57]. Diffraction data for the wild-type protein were collected in-house using a Bruker MicroStar rotating anode and processed with SAINT [58]. All CAP mutant data were collected at the Diamond Light Source beams I-04 and I-24 and processed using Mosflm [59] and Scala [60]. CAP structures were solved using molecular replacement with Phaser [61] using CAP (PDB 1I5Z). Model building and refinement were accomplished iteratively using COOT [62] and Refmac5 [63] in CCP4 [59]. CAP structures from crystals produced at pH 6.5 were indistinguishable from those previously produced at pH 7.5 [64]. Structural and refinement statistics are provided in Table S4. Full details of GlxR crystallography and analysis of the structures will be reported elsewhere [45]. Members of the CAP family often crystallise with more than one protein chain in the asymmetric unit. In these cases the functional protein dimer is either generated by the crystallographic 2-fold axis on each of the protein chains or by noncrystallographic symmetry leading to a varying degree of asymmetry [65],[66]. Significantly different conformations for each monomer have been observed in some homodimeric bacterial regulator proteins, most notably Mt-CRP [67]. The structures presented here contain one dimer (wild-type CAP in space group P2_1_), two dimers (wild-type in space group P1), and three dimers (V140A CAP in space group I2) (see Table S4). In all cases the dimers are symmetric with no significant differences between the two protein chains than for the functional dimer.

### Coarse-Grained Simulations

ENM simulations were performed using our own code based on the regular implementation [31],[68]. The spring constants were set to a constant value of 1 kcal mol^−1^ Å^−2^ with a cutoff radius of 8 Å, and only the Cα atoms in the protein were considered. The presence of cAMP effector at the binding site was treated by the addition of one node at the mass weighted average coordinate for each ligand. Varying the spring constant of any springs attached to a single residue of the protein was used to represent side chain mutations. The allosteric free energy was calculated by summing over modes 1 to *n*. *n* was determined by examining where values *K*_2_/*K*_1_ converged (Figure S8). The final results quoted used the converged value of *K*_2_/*K*_1_. PDB files for constructing CAP ENMs were 1CGP, 1G6N, 1HW5, 1I5Z, 1I6X, 1J59, 1O3T, 1RUN, 1RUO, 1ZRC, 1ZRD, 1ZRF, 2GZW, 4HZF (this work), and an additional in-house file isostructural to 2GZW. The PDB file for constructing the GlxR ENM was 3R6S.

### Super-Coarse-Grained Model

The CAP and GlxR proteins were modelled as two blocks for each monomer, one for the ligand binding domain and one for the DNA binding domain. We assigned one internal breathing mode to each subunit and allowed each subunit to move, producing seven degrees of freedom. For the apo-protein the internal subunit coupling strengths are characterized by *k*_1_ though *k*_4_ and the intersubunit couplings by *k*_12_, *k*_13_, and *k*_24_ (Figure 4b). The effect of one ligand binding was included by modifying *k*_1_ by a factor β, *k*_12_ by α, and *k*_12_ by γ. The second ligand binding was therefore represented by further modifying *k*_2_ by β, *k*_12_ by a further factor of α, and *k*_24_ by γ. The allosteric free energy was determined from the determinant of the interaction matrix [69]. The couplings were defined from PCA analysis of 300 ns molecular dynamics simulations for the three states. In each case the protein was divided into the four zones by performing a rotational-translational-block approximation (Figure 8a) [46],[70]. Examination of the couplings calculated for each of the three states allowed calculation of the apo values and the ligand binding factors. Varying the values of *k*_1_, *k*_2_, and *k*_12_ represents mutations in residues affecting the intra- and the interblock interactions. Wild-type values for CAP are: *k*_1_ = *k*_2_ = 13.70, *k*_12_ = 27.08, *k*_3_ = *k*_4_ = 3.98, *k*_13_ = *k*_24_ = 5.19 kcal mol^−1^ Å^−2^, α = 1.30, β = 0.560, and γ = 0.901. Wild-type values for GlxR are: *k*_1_ = *k*_2_ = 12.85, *k*_12_ = 24.67, *k*_3_ = *k*_4_ = 3.98, *k*_13_ = *k*_24_ = 4.21 kcal mol^−1^ Å^−2^, α = 1.40, β = 0.71, and γ = 0.99.

### Atomistic Simulation

Molecular dynamics (MD) simulations employed the harmonic force field equations used in the ff99SB and GAFF force fields within the AMBER simulation program [71].
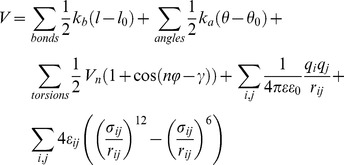
The simulations employed the ff99SB force field for the CAP protein and the GAFF force field (v. 1.4) for cAMP. ff99SB force field is used as the energetic interactions of side chains, which are reasonably represented by this force field [72], and outperforms the ff03 force field [73]. MD calculations used a short-range cutoff of 10 Å, with the long-range portion of the Coulomb potential represented by an Ewald summation, and employed a time step of 2 fs. The bond lengths were constrained by the SHAKE algorithm. The initial starting structures were obtained directly from X-ray diffraction. These structures were then solvated in TIP3P water and energy minimized prior to simulation [74]. The system was heated to 300 K over a period of 20 ps and further equilibrated for 40 ns. Production runs at 300 K were carried out over 200 ns. PCA was performed by diagonalising the mass weighted covariance matrix of the atomistic simulations. The eigenvectors represent the shape of the atomistic motion and the corresponding eigenvalues the extent of the motion.

### Analysis of CAP Variants

To determine if Δ*K*_2_/*K*_1_, hereon denoted *x*, is associated with the mutation rate of amino acids, we first estimated the relative amino acid mutation rate using the sequence data for CAP variants and we then statistically tested for an effect of *x* on this rate. Relative mutation rate was estimated by finding the minimum number of amino acid mutations needed to generate the observed variations in the sequence data, which we denote *N*. For each of the 165 proteins we found the protein having the smallest number of amino acid differences. The sum of these differences gave *N*. When summing differences, if more than one protein had the minimum difference, we included all the proteins having the minimum. We then determined the number of these mutations that were associated with each of the 210 amino acids, which we denote *n_i_*. Thus, *n_i_* estimates the relative mutation rate of amino acid *i*, and these estimates account for the evolutionary history of the proteins. If all amino acids had an equal mutation rate, then we would expect the *n_i_* to all be approximated by *N*/210. We assumed that the true relative rate of mutation was related to *x* according to the logistic function: *μ*(*x*) = 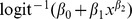, where *β*_0_, *β*_1_, and *β*_2_ are constants. To account for overdispersion among the *n_i_*, which might be due to unmeasured covariates associated with the proteins, we assumed that the variation between the *n_i_* could be described by the beta-binomial distribution. Under these assumptions, the log-likelihood of the model described by the set of parameters **θ** = {*β*_0_,*β*_1_,*β*_2_,*φ*}, is given by:
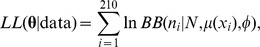
where *BB*(*n*|*N*,*μ*,*φ*) is the beta-binomial distribution, which describes the probability of observing *n* successes from *N* trials when, on average, successes occur with probability *μ* and variation in this probability among replicates is described by the beta-distribution with variance *μ*(1−*μ*)*φ*/(1+*φ*) [75].

Evidence that mutation rate was related to *x* was found by applying a likelihood ratio test (LRT) comparing the fit of the full model with the model that ignored *x* (i.e., when *β*_1_ = *β*_2_ = 0). Let *LL*_1_ and *LL*_0_ be the maximum log-likelihood of the full model and the simpler model, respectively. Under the null hypothesis that *x* is not associated with mutation rate, the test statistic *G* = 2[*LL*_1_−*LL*_0_] is chi-square distributed with two degrees of freedom, as the more complex model has two additional free parameters: *β*_1_ and *β*_2_. A LRT was also used to test for overdispersion by comparing the fit from the full model described above with the model that assumed variation had a binomial distribution (*φ* is vanishingly small). This latter test, if significant, justifies the use of the beta-binomial distribution rather than the binomial. Confidence intervals for model parameters were estimated using the likelihood profile approach.

The genome accession numbers analysed are: NP_232242.1, NP_246094.1, NP_249343.1, NP_439118.1, NP_458435.1, NP_462369.1, NP_671249.1, NP_716257.1, NP_760245.1, NP_799172.1, NP_873260.1, NP_927748.1, YP_052151.1, YP_089126.1, YP_128534.1, YP_152459.1, YP_205663.1, YP_237645.1, YP_262678.1, YP_272974.1, YP_455981.1, YP_492074.1, YP_526229.1, YP_564189.1, YP_588978.1, YP_606222.1, YP_690711.1, YP_693743.1, YP_718344.1, YP_751967.1, YP_855526.1, YP_928876.1, YP_941848.1, YP_960806.1, YP_001048976.1, YP_001092716.1, YP_001143048.1, YP_001178491.1, YP_001189422.1, YP_001218107.1, YP_001343325.1, YP_001440391.1, YP_001443362.1, YP_001464812.1, YP_001475605.1, YP_001503357.1, YP_001675803.1, YP_001759053.1, YP_001909102.1, YP_002152521.1, YP_002228709.1, YP_002294894.1, YP_002476451.1, YP_002650381.1, YP_002801694.1, YP_002875051.1, YP_002893931.1, YP_002923696.1, YP_002986005.1, YP_003002662.1, YP_003008634.1, YP_003039145.1, YP_003074496.1, YP_003255073.1, YP_003261368.1, YP_003469961.1, YP_003532766.1, YP_003555253.1, YP_003812150.1, YP_003914673.1, YP_004117516.1, YP_004211044.1, YP_004382110.1, YP_004391469.1, YP_004419866.1, YP_004472683.1, YP_004565203.1, YP_004713013.1, YP_004821770.1, YP_005091541.1, YP_005334361.1, YP_005458526.1, YP_005817463.1, YP_006006755.1, YP_006238931.1, YP_006286710.1, YP_006326252.1, YP_006459298.1, YP_006523113.1, YP_006588319.1, ZP_00134303.1, ZP_00991497.1, ZP_01161654.1, ZP_01215522.1, ZP_01815379.1, ZP_01894180.1, ZP_01898714.1, ZP_02478644.1, ZP_02958582.1, ZP_03319669.1, ZP_03611762.1, ZP_03825776.1, ZP_04636540.1, ZP_04640765.1, ZP_04752629.1, ZP_04977551.1, ZP_05043634.1, ZP_05637197.1, ZP_05774479.1, ZP_05849758.1, ZP_05879825.1, ZP_05880998.1, ZP_05919259.1, ZP_05972068.1, ZP_05990699.1, ZP_06018230.1, ZP_06051220.1, ZP_06126446.1, ZP_06542208.1, ZP_06637662.1, ZP_07161146.1, ZP_07222409.1, ZP_07266238.1, ZP_07379670.1, ZP_07395486.1, ZP_07528968.1, ZP_07744420.1, ZP_07777878.1, ZP_07888842.1, ZP_08039455.1, ZP_08068248.1, ZP_08079426.1, ZP_08100561.1, ZP_08148040.1, ZP_08310711.1, ZP_08519301.1, ZP_08725568.1, ZP_08731411.1, ZP_08745737.1, ZP_08754750.1, ZP_09013912.1, ZP_09039716.1, ZP_09185001.1, ZP_09505069.1, ZP_09557915.1, ZP_09710329.1, ZP_09778630.1, ZP_09972449.1, ZP_10075284.1, ZP_10125383.1, ZP_10128956.1, ZP_10135899.1, ZP_10142323.1, ZP_10146384.1, ZP_10426764.1, ZP_10438900.1, ZP_10622342.1, ZP_10628430.1, ZP_10630449.1, ZP_10643899.1, ZP_10655392.1, ZP_10677933.1, ZP_10700164.1, and ZP_10763153.1.

## Supporting Information

Figure S1 **ENM representation of CAP.** Alpha helices are represented in magenta and beta sheets in yellow. Blue spheres show the positions of the Cα atoms, and the black lines display the connectivity of the Hookean springs with a cutoff of 8 Å. Apo and singly bound ENMs were constructed by manually removing cAMP from the holoenzyme.
(TIF)

Figure S2 **Validation of ENM methodology.** (A) CAP B-factors are independent of coarse-grained methodology. The chart represents the B-factor plotted against amino acid number for the crystal structure, ENM, and molecular dynamics. (B) Mode frequencies are independent of methodology. The chart represents the mode frequency plotted against mode number for ENM and molecular dynamics.
(TIF)

Figure S3 **ENM predicted residue interactions that impact on cooperativity.** (A) The change in cooperativity that occurs when *k*_R_/*k* is varied at the indicated residue (legend) against every amino acid within the same monomer (within an 8 Å cutoff). (B) The change in cooperativity that occurs when *k*_R_/*k* is varied at the indicated residue (legend) against every amino acid within the opposing monomer (within an 8 Å cutoff).
(TIF)

Figure S4 **Least-squares superposition of one representative chain of each of the seven doubly cAMP-bound crystal structures treating the two domains (dimerization/cAMP-binding domain and DNA-binding domain) as rigid bodies with a flexible linker (wild-type, green; V132A, cyan; V132L, dark cyan; V140A, magenta; V140L, orange; H160L, red).** The transformation matrices were obtained using RAPIDO [76].
(TIF)

Figure S5 **Fitting of ITC data.** Binding isotherm for a representative data set for the calorimetric titration of cAMP to wild-type CAP protein showing experimental data and fitted curves for two and three molecules of ligand cAMP. The inset shows the structure of CAP (green) with three bound molecules of cAMP (blue).
(TIF)

Figure S6 **Calculated and observed values for cooperativity in CAP.** (A) The ratio of the second to first dissociation constants for cAMP (*K*_2_/*K*_1_) for wild-type and mutant CAP proteins were calculated from the ENMs (calculated) or obtained by ITC (observed). The coloured lines correspond to the value for *K*_2_/*K*_1_ in the wild-type to enable comparison of the direction of change. (B) Values for *K*_2_/*K*_1_ obtained by ITC plotted against values for *K*_2_/*K*_1_ predicted by the ENM demonstrating the correlation between the extents of experimentally observed and predicted values for *K*_2_/*K*_1_. Dotted line represents the 95% confidence interval for the linear regression (R^2^ = 0.85).
(TIF)

Figure S7 **Mapping local dynamics in CAP.** (A) The effect of mutation of V140 and H160 on local dynamics over the CAP monomer. The chart represents the percentage variation in B-factor from the wild-type ENM plotted against amino acid number. Inset shows the same chart with an expansion of the *y*-axis. (B) The chart is identical to that shown in panel C except with the *y*-axis expanded.
(TIF)

Figure S8 **The dependence of** ***K*** **_2_/** ***K*** **_1_ on the number of summed modes.** The chart represents the calculated value for *K*_2_/*K*_1_ from the ENM plotted against the total number of summed modes.
(TIF)

Table S1 **Least-squares superposition of all independent protein chains in each of the doubly cAMP-bound CAP crystal structures.**
(PDF)

Table S2 **Experimental thermodynamic parameters for CAP proteins.**
(PDF)

Table S3 **Experimental thermodynamic parameters for GlxR proteins.**
(PDF)

Table S4 **Crystallographic data collection and refinement statistics.**
(PDF)

## Abbreviations

ANOVA: analysis of variance
ATP: adenosine triphosphate
cAMP: 3′-5′-cyclic adenosine monophosphate
CAP: Catabolite Activator Protein
CI: confidence interval
CRP/FNR: cAMP receptor protein/fumarate and nitrate reduction regulator
DNA: deoxyribonucleic acid
ENM: elastic network model
IPTG: isopropyl β-D-1-thiogalactopyranoside
ITC: isothermal calorimetry
LRT: likelihood-ratio test
MD: molecular dynamics
NMR: nuclear magnetic resonance
PCA: principal component analysis
RMSD: root-mean-square deviation
RNA: ribonucleic acid
SEM: standard error of the mean
