# Supplementary figures and images for: Modulation of Global Low-Frequency Motions Underlies Allosteric Regulation: Demonstration in CRP/FNR Family Transcription Factors
Source: PLoS Biol. 2013 Sep 10;11(9):e1001651. doi: 10.1371/journal.pbio.1001651 (PMC3769225; doi:10.1371/journal.pbio.1001651)

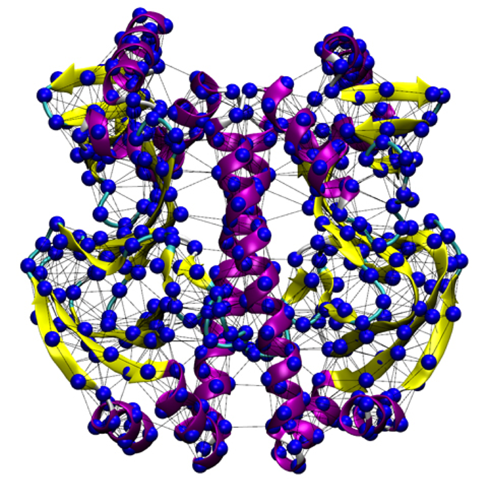

Supplement: Figure S1 — ENM representation of CAP. Alpha helices are represented in magenta and beta sheets in yellow. Blue spheres show the positions of the Cα atoms, and the black lines display the connectivity of the Hookean springs with a cutoff of 8 Å. Apo and singly bound ENMs were constructed by manually removing cAMP from the holoenzyme. (TIF) [file pbio.1001651.s001.tif]

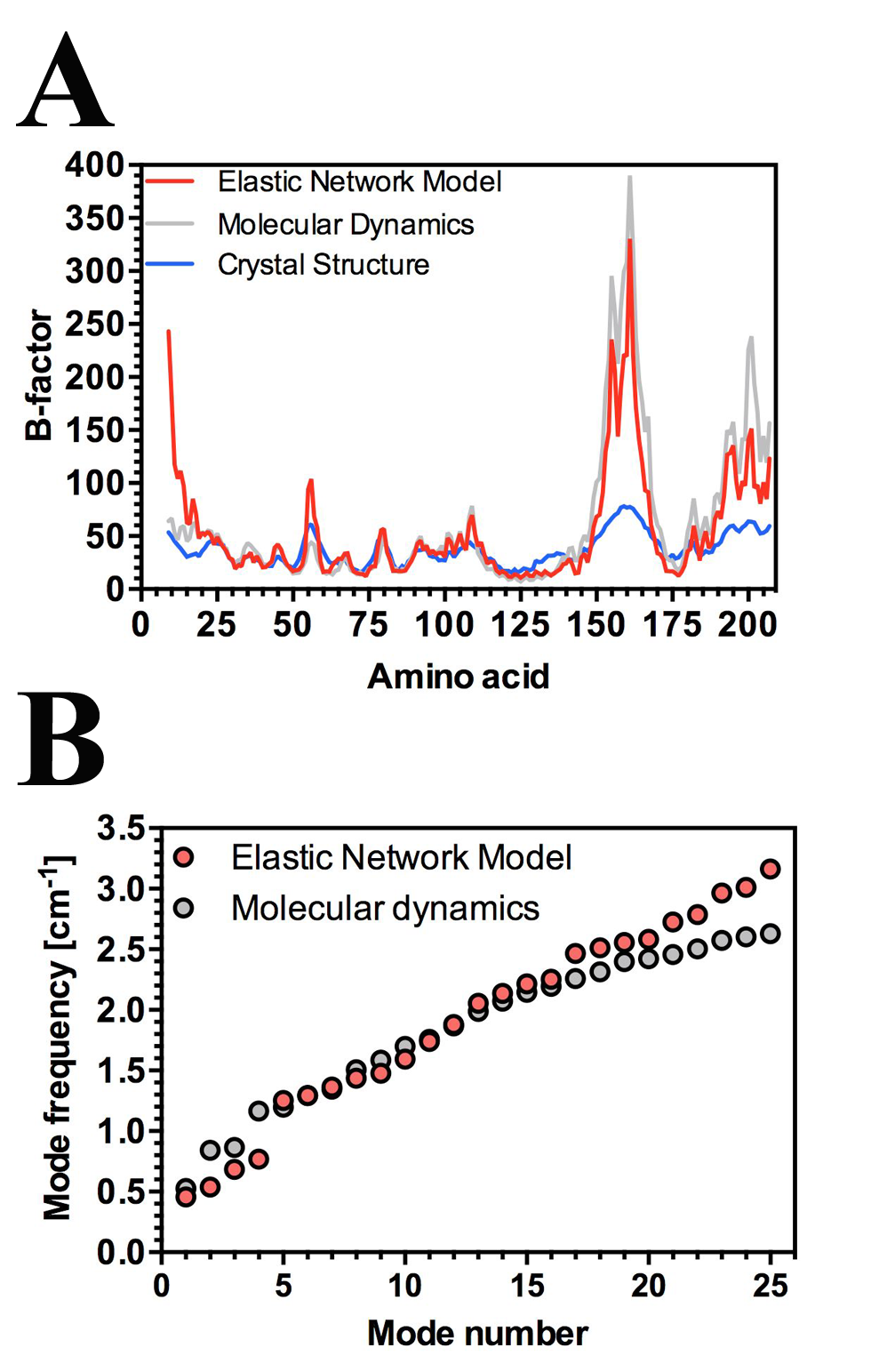

Supplement: Figure S2 — Validation of ENM methodology. (A) CAP B-factors are independent of coarse-grained methodology. The chart represents the B-factor plotted against amino acid number for the crystal structure, ENM, and molecular dynamics. (B) Mode frequencies are independent of methodology. The chart represents the mode frequency plotted against mode number for ENM and molecular dynamics. (TIF) [file pbio.1001651.s002.tif]

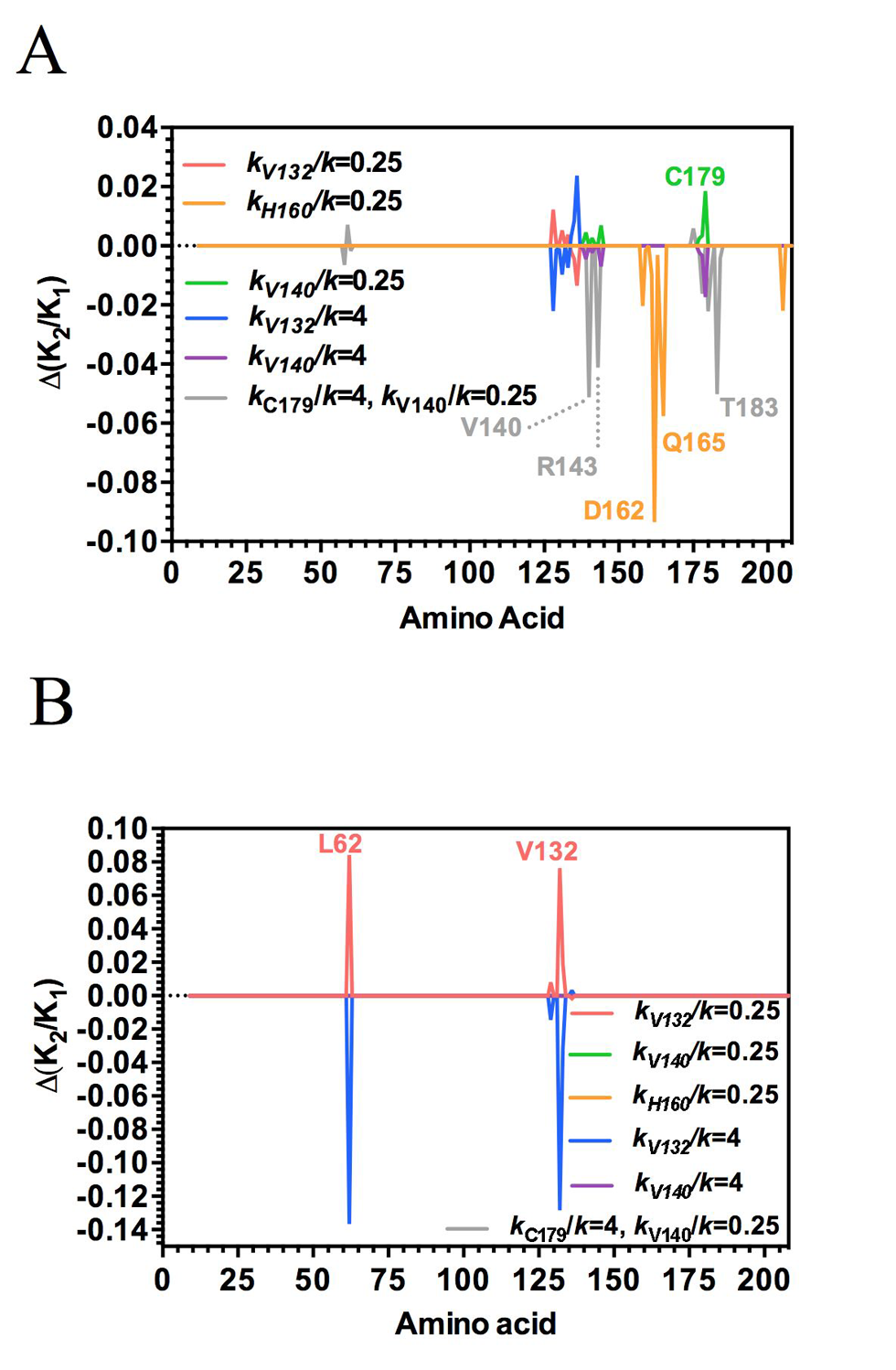

Supplement: Figure S3 — ENM predicted residue interactions that impact on cooperativity. (A) The change in cooperativity that occurs when k R/k is varied at the indicated residue (legend) against every amino acid within the same monomer (within an 8 Å cutoff). (B) The change in cooperativity that occurs when k R/k is varied at the indicated residue (legend) against every amino acid within the opposing monomer (within an 8 Å cutoff). (TIF) [file pbio.1001651.s003.tif]

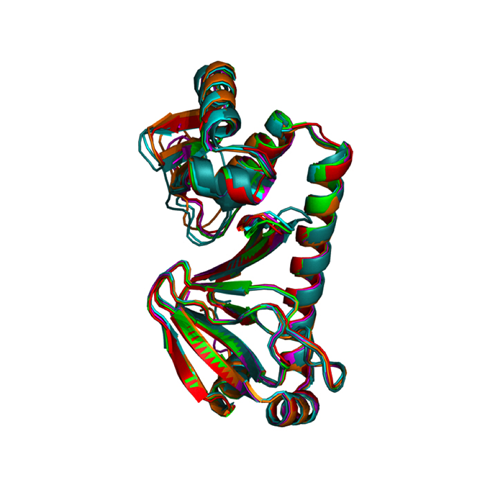

Supplement: Figure S4 — Least-squares superposition of one representative chain of each of the seven doubly cAMP-bound crystal structures treating the two domains (dimerization/cAMP-binding domain and DNA-binding domain) as rigid bodies with a flexible linker (wild-type, green; V132A, cyan; V132L, dark cyan; V140A, magenta; V140L, orange; H160L, red). The transformation matrices were obtained using RAPIDO [76]. (TIF) [file pbio.1001651.s004.tif]

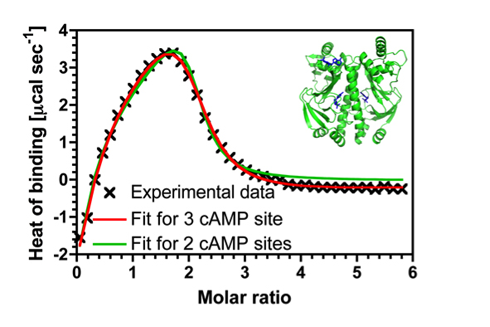

Supplement: Figure S5 — Fitting of ITC data. Binding isotherm for a representative data set for the calorimetric titration of cAMP to wild-type CAP protein showing experimental data and fitted curves for two and three molecules of ligand cAMP. The inset shows the structure of CAP (green) with three bound molecules of cAMP (blue). (TIF) [file pbio.1001651.s005.tif]

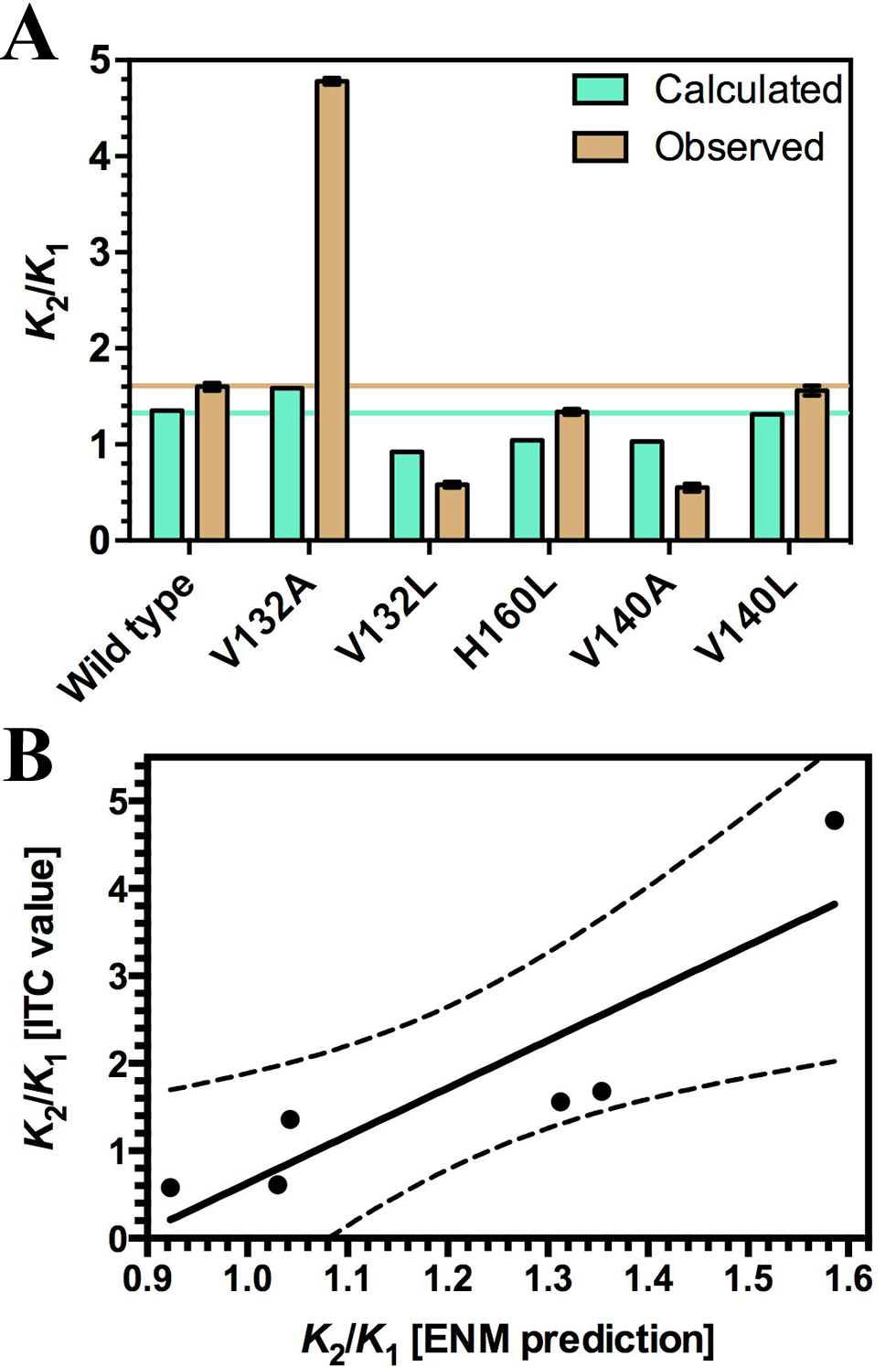

Supplement: Figure S6 — Calculated and observed values for cooperativity in CAP. (A) The ratio of the second to first dissociation constants for cAMP (K 2/K 1) for wild-type and mutant CAP proteins were calculated from the ENMs (calculated) or obtained by ITC (observed). The coloured lines correspond to the value for K 2/K 1 in the wild-type to enable comparison of the direction of change. (B) Values for K 2/K 1 obtained by ITC plotted against values for K 2/K 1 predicted by the ENM demonstrating the correlation between the extents of experimentally observed and predicted values for K 2/K 1. Dotted line represents the 95% confidence interval for the linear regression (R2 = 0.85). (TIF) [file pbio.1001651.s006.tif]

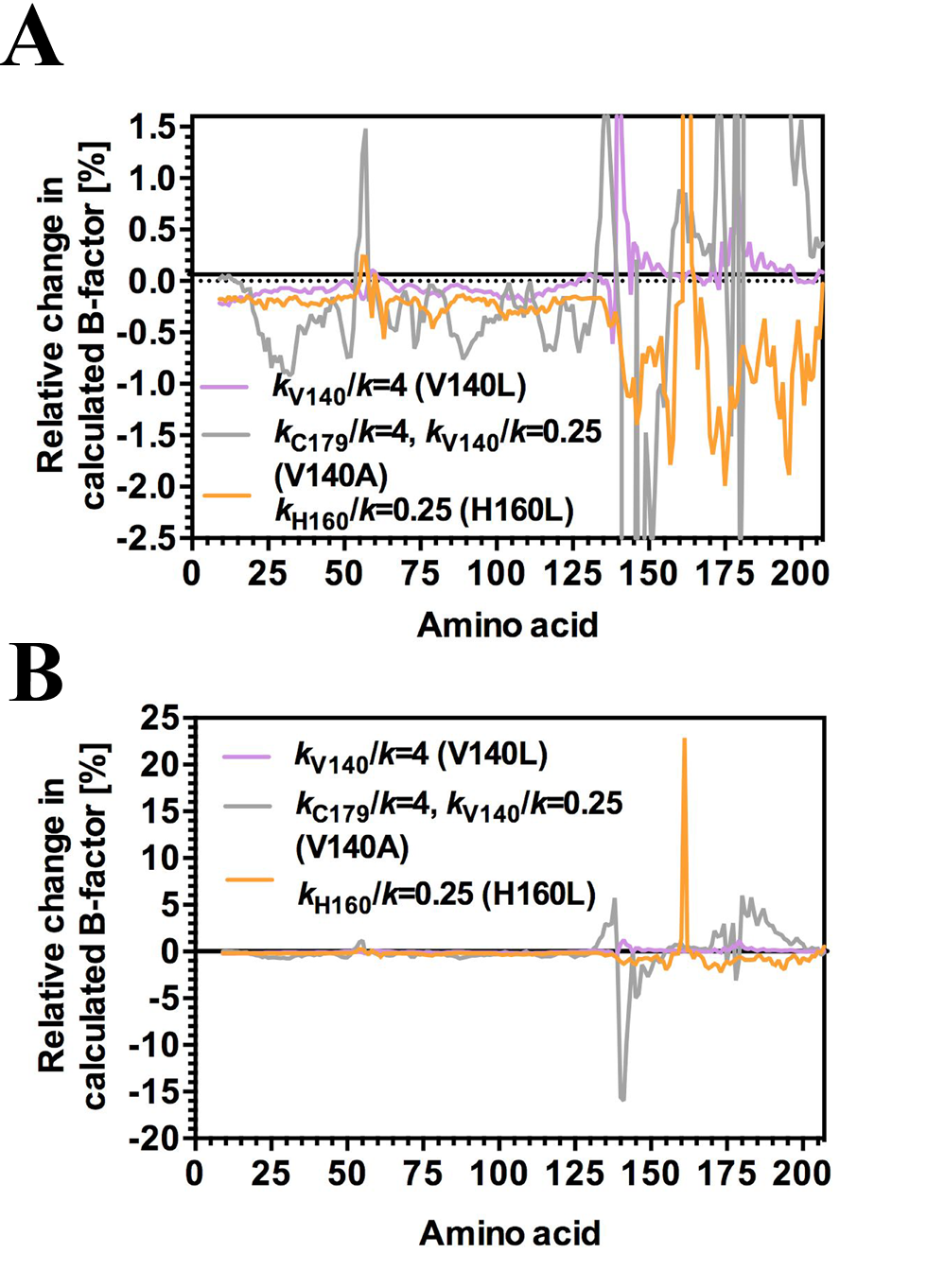

Supplement: Figure S7 — Mapping local dynamics in CAP. (A) The effect of mutation of V140 and H160 on local dynamics over the CAP monomer. The chart represents the percentage variation in B-factor from the wild-type ENM plotted against amino acid number. Inset shows the same chart with an expansion of the y-axis. (B) The chart is identical to that shown in panel C except with the y-axis expanded. (TIF) [file pbio.1001651.s007.tif]

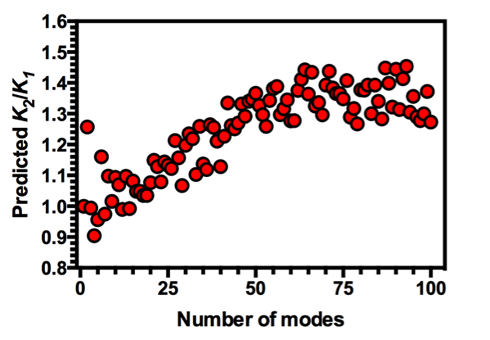

Supplement: Figure S8 — The dependence of K 2/ K 1 on the number of summed modes. The chart represents the calculated value for K 2/K 1 from the ENM plotted against the total number of summed modes. (TIF) [file pbio.1001651.s008.tif]
