# Supplementary material for: Modulation of Global Low-Frequency Motions Underlies Allosteric Regulation: Demonstration in CRP/FNR Family Transcription Factors
Source: PLoS Biol. 2013 Sep 10;11(9):e1001651. doi: 10.1371/journal.pbio.1001651 (PMC3769225; doi:10.1371/journal.pbio.1001651)
Supplement: Table S1 — Least-squares superposition of all independent protein chains in each of the doubly cAMP-bound CAP crystal structures. (PDF) [file pbio.1001651.s009.pdf]

Table S1. Least-squares superposition of all independent protein chains in each of the doubly cAMP-bound CAP crystal structures

	wt A	wt B	wt C	wt D	V132A A	V132A B	V132L A	V132L B	V140A A	V140A B	V140A C	V140A D	V140A E	V140A F	V140L A	V140L B	H160L A	H160L B
wt A	n/a	0.51	0.72	0.49	0.68	0.54	*0.95*	0.51	0.62	0.72	0.76	0.64	0.77	0.66	*0.78*	0.61	0.61	0.72
wt B		n/a	0.63	*0.48*	*0.64*	0.57	*0.86*	0.58	*0.54*	*0.59*	*0.68*	*0.56*	*0.66*	*0.67*	*0.71*	0.74	0.59	0.71
wt C			n/a	*0.60*	*0.63*	*063*	*0.84*	0.72	*0.55*	*0.51*	*0.78*	*0.53*	*0.69*	*0.71*	*0.61*	0.75	0.66	*0.88*
wt D				n/a	*0.56*	0.54	*0.83*	0.60	*0.44*	*0.49*	*0.67*	*0.46*	*0.66*	*0.62*	*0.68*	0.71	0.51	0.74
V132A A					n/a	0.53	*0.92*	0.68	*0.58*	*0.55*	*0.71*	*0.61*	*0.65*	*0.70*	*0.60*	0.82	0.72	0.92
V132A B						n/a	0.67	0.81	0.47	0.46	0.60	0.48	0.58	0.61	0.51	*0.57*	0.54	0.48
V132L A							n/a	0.60	*0.77*	0.77	*0.53*	*0.76*	*0.48*	*0.69*	*0.89*	*0.81*	0.66	0.91
V132L B								n/a	0.62	0.60	0.56	0.53	0.55	0.56	0.67	*0.92*	*1.12*	0.52
V140A A									n/a	*0.31*	*0.65*	*0.24*	*0.63*	*0.60*	*0.64*	0.65	0.54	*0.78*
V140A B										n/a	*0.65*	*0.30*	*0.62*	*0.67*	*0.66*	0.69	0.55	0.76
V140A C											n/a	*0.65*	*0.19*	*0.34*	*0.82*	0.84	0.66	*0.74*
V140A D												n/a	*0.63*	*0.62*	*0.67*	0.66	0.58	0.66
V140A E													n/a	*0.43*	*0.77*	0.80	0.68	0.75
V140A F														n/a	0.86	0.82	0.65	*0.62*
V140L A															n/a	0.54	0.66	0.92
V140L B																n/a	0.75	0.66
H160L A																	n/a	0.56

All structures were aligned with the flexible two-domain model (dimerization/cAMP-binding domain and DNA-binding domain) using the RAPIDO web-server [76]. The values in the table correspond to flexible *root-mean square deviations* (rmsd) over all Cα-atoms in both domains. The capital letters refer to the independent chain in each crystal structure. The rmsd's for different chains of the same crystal structure are similar to the values between the different CAP variants. The numbers printed in italics correspond to a pair of chains that are so similar that they are treated as one domain only.
