# Supplementary material for: Modulation of Global Low-Frequency Motions Underlies Allosteric Regulation: Demonstration in CRP/FNR Family Transcription Factors
Source: PLoS Biol. 2013 Sep 10;11(9):e1001651. doi: 10.1371/journal.pbio.1001651 (PMC3769225; doi:10.1371/journal.pbio.1001651)
Supplement: Table S2 — Experimental thermodynamic parameters for CAP proteins. (PDF) [file pbio.1001651.s010.pdf]

Table S2. Experimental thermodynamic parameters for CAP proteins.

CAP Protein	ΔH_1 (kcal mol^{-1})	ΔH_2 (kcal mol^{-1})	ΔG_1 (kcal mol^{-1})	ΔG_2 (kcal mol^{-1})	$-T\Delta S_1$ (kcal mol^{-1})	$-T\Delta S_2$ (kcal mol^{-1})
Wild type	-2.0 ±0.1 (32)	8.2 ±0.2 (32)	-6.9 ±0.0 (32)	-6.6 ±0.0 (32)	-4.9 ±0.1 (32)	-14.8 ±0.2 (32)
V132A	-2.7 ±0.3 (20)	0.7 ±0.2 (20)	-7.0 ±0.1 (20)	-6.1 ±0.1 (20)	-3.9 ±0.5 (20)	-7.0 ±0.3 (20)
V132L	-1.9 ±0.1 (17)	6.1 ±0.1 (17)	-7.3 ±0.0 (17)	-7.7 ±0.0 (17)	-5.5 ±0.1 (17)	-13.7 ±0.1 (17)
H160L	-2.9 ±0.1 (31)	9.8 ±0.1 (31)	-6.8 ±0.0 (31)	-6.6 ±0.0 (31)	-3.9 ±0.1 (31)	-16.4 ±0.1 (31)
V140A	-4.6 ±0.3 (29)	12.3 ±0.4 (29)	-6.8 ±0.0 (29)	-7.2 ±0.0 (29)	-2.3 ±0.4 (29)	-19.9 ±0.4 (29)
V140L	-2.2 ±0.1 (27)	7.2 ±0.1(27)	-7.2 ±0.1 (27)	-6.9 ±0.1 (27)	-5.0 ±0.1 (27)	-14.1 ±0.1 (27)

Mean values ± S.E.M. are given for wild type and mutant CAP for the first and second cAMP binding events. *n* is provided in parentheses.
