# Supplementary material for: Modulation of Global Low-Frequency Motions Underlies Allosteric Regulation: Demonstration in CRP/FNR Family Transcription Factors
Source: PLoS Biol. 2013 Sep 10;11(9):e1001651. doi: 10.1371/journal.pbio.1001651 (PMC3769225; doi:10.1371/journal.pbio.1001651)
Supplement: Table S3 — Experimental thermodynamic parameters for GlxR proteins. (PDF) [file pbio.1001651.s011.pdf]

Table S3. Experimental thermodynamic parameters for GlxR proteins.

CAP Protein	ΔH_1 (kcal mol^{-1})	ΔH_2 (kcal mol^{-1})	ΔG_1 (kcal mol^{-1})	ΔG_2 (kcal mol^{-1})	$-T\Delta S_1$ (kcal mol^{-1})	$-T\Delta S_2$ (kcal mol^{-1})
Wild type	2.3 ±0.0 (18)	2.8 ±0.2(18)	-7.4 ±0.0 (18)	-5.7 ±0.0 (18)	-9.7 ±0.1 (18)	-8.5 ±0.1 (18)
L134V	4.8 ±0.1 (26)	2.5 ±0.1 (26)	-6.1 ±0.0 (26)	-5.3 ±0.0 (26)	-10.9 ±0.1 (26)	-7.8 ±0.1 (26)
A131V	-3.2 ±0.1 (30)	1.5 ±0.1 (30)	-6.5 ±0.1 (30)	-5.7 ±0.1 (30)	-3.3 ±0.1 (30)	-7.1 ±0.1 (30)

Mean values ± S.E.M. are given for wild type and mutant GlxR for the first and second cAMP binding events. *n* is provided in parentheses.
