# Supplementary material for: Modulation of Global Low-Frequency Motions Underlies Allosteric Regulation: Demonstration in CRP/FNR Family Transcription Factors
Source: PLoS Biol. 2013 Sep 10;11(9):e1001651. doi: 10.1371/journal.pbio.1001651 (PMC3769225; doi:10.1371/journal.pbio.1001651)
Supplement: Table S4 — Crystallographic data collection and refinement statistics. (PDF) [file pbio.1001651.s012.pdf]

Table S4. Crystallographic data collection and refinement statistics.

**Data Collection**							
Protein	WT	WT	V132A	V132L	V140A	V140L	H160L
X-ray source	In house[d]	I24[c]	I04[c]	SLS	I04[c]	I24[c]	I24[c]
Wavelength (Å)	1.5418	0.9795	0.9687	0.9795	0.9687	0.9795	0.9687
Space group	P1	$P12_11$	$P2_12_12_1$	$P2_12_12_1$	I121	$P2_12_12_1$	$P12_11$
Cell dimensions							
a, b, c (Å)	45.3, 57.1, 91.4	45.5, 101.9, 53.8	45.9, 93.3, 104.2	50.3, 93.1, 105.4	291.3, 50.2, 116.3	45.4, 99.8, 102.8	56.8, 72.3, 58.4
α, β, γ (º)	91.3, 102.0, 104.2	90.0, 111.1, 90.0	90.0, 90.0, 90.0	90.0, 90.0, 90.0	90.0, 109.0, 90.0	90.0, 90.0, 90.0	90.0, 109.9, 90.0
Resolution (Å)	2.75	1.48	2.20	2.05	1.75	2.30	1.50
R_{merge} (%)[a,b]	0.077 (0.40)	0.062 (0.425)	0.084 (0.228)	0.076 (0.524)	0.075 (0.366)	0.116 (0.642)	0.054 (0.451)
I/σ(I)[a]	12.6 (2.0)	8.0 (2.0)	11.5 (4.5)	15.7 (4.3)	12.2 (3.7)	6.3 (2.4)	6.9 (2.3)
Completeness (%)[a]	91.2	98.8 (99.7)	96.5 (83.0)	99.3 (98.8)	99.6 (98.9)	99.4 (99.1)	99.7 (99.7)
Multiplicity[a]	3.9	3.0 (3.0)	5.5 (3.4)	7.9 (8.2)	5.4 (5.1)	4.9 (4.7)	3.3 (3.2)
**Refinement**							
Resolution (Å)	2.75	1.48	2.20	2.05	1.75	2.30	1.50
No. measurements	85778	226403	128654	233799	858471	103642	233544
No. unique	22122	71204	23348	29362	160426	21298	70844
R_{work} / R_{free}	0.1855 / 0.2659	0.195 / 0.2415	0.1787 / 0.2332	0.2008 / 0.2563	0.1848 / 0.2157	0.195 / 0.2619	0.1803 / 0.2083
No. protein chains	4	2	2	2	6	2	2
No. atoms							
Protein	6012	3124	3165	3165	9507	3109	3196
Ligand/ion	84	109	69	42	189	64	63
Water	112	293	116	124	927	83	331
Average B-factor	31.089	30.662	32.739	43.22	24.386	48.431	23.915
R.m.s. deviations							
Bond lengths (Å)	0.0127	0.0112	0.0189	0.0194	0.0114	0.015	0.0115
Bond angles (º)	0.9048	1.6357	1.0213	1.0192	1.5114	1.9485	1.6006
PDB codes	e	4HZF	4I0A	4I09	4I02	4I01	4I0B

[a] Number in parentheses is for the last shell
[b] $R = \sum(\mathrm{Abs}(I - \langle I \rangle) / \sum(I))$
[c] Diamond Light Source
[d] Bruker MicroStar
[e] Isostructural to 2GZW
